# Microbial production of high octane and high sensitivity olefinic ester biofuels

**DOI:** 10.1186/s13068-023-02301-7

**Published:** 2023-04-04

**Authors:** David N. Carruthers, Jinho Kim, Daniel Mendez-Perez, Eric Monroe, Nick Myllenbeck, Yuzhong Liu, Ryan W. Davis, Eric Sundstrom, Taek Soon Lee

**Affiliations:** 1 Biological Systems and Engineering Division, Lawrence Berkeley National Laboratory, Berkeley, CA 94720 USA; 2 Joint BioEnergy Institute, 5885 Hollis Street, Emeryville, CA 94608 USA; 3 Sandia National Laboratories, Livermore, CA 94551 USA; 4 Advanced Biofuels and Bioproducts Process Development Unit, Emeryville, CA 94608 USA

**Keywords:** Olefinic esters, Isoprenyl acetate, Prenyl acetate, IPP-bypass pathway, Fuel blends, MVA pathway, Alcohol acyltransferase, Isoprenyl lactate, Prenyl lactate

## Abstract

**Background:**

Advanced spark ignition engines require high performance fuels with improved resistance to autoignition. Biologically derived olefinic alcohols have arisen as promising blendstock candidates due to favorable octane numbers and synergistic blending characteristics. However, production and downstream separation of these alcohols are limited by their intrinsic toxicity and high aqueous solubility, respectively. Bioproduction of carboxylate esters of alcohols can improve partitioning and reduce toxicity, but in practice has been limited to saturated esters with characteristically low octane sensitivity. If olefinic esters retain the synergistic blending characteristics of their alcohol counterparts, they could improve the bioblendstock combustion performance while also retaining the production advantages of the ester moiety.

**Results:**

Optimization of *Escherichia coli* isoprenoid pathways has led to high titers of isoprenol and prenol, which are not only excellent standalone biofuel and blend candidates, but also novel targets for esterification. Here, a selection of olefinic esters enhanced blendstock performance according to their degree of unsaturation and branching. *E. coli* strains harboring optimized mevalonate pathways, thioester pathways, and heterologous alcohol acyltransferases (ATF1, ATF2, and SAAT) were engineered for the bioproduction of four novel olefinic esters. Although prenyl and isoprenyl lactate titers were limited to 1.48 ± 0.41 mg/L and 5.57 ± 1.36 mg/L, strains engineered for prenyl and isoprenyl acetate attained titers of 176.3 ± 16.0 mg/L and 3.08 ± 0.27 g/L, respectively. Furthermore, prenyl acetate (20% bRON = 125.8) and isoprenyl acetate (20% bRON = 108.4) exhibited blend properties comparable to ethanol and significantly better than any saturated ester. By further scaling cultures to a 2-L bioreactor under fed-batch conditions, 15.0 ± 0.9 g/L isoprenyl acetate was achieved on minimal medium. Metabolic engineering of acetate pathway flux further improved titer to attain an unprecedented 28.0 ± 1.0 g/L isoprenyl acetate, accounting for 75.7% theoretical yield from glucose.

**Conclusion:**

Our study demonstrated novel bioproduction of four isoprenoid oxygenates for fuel blending. Our optimized *E. coli* production strain generated an unprecedented titer of isoprenyl acetate and when paired with its favorable blend properties, may enable rapid scale-up of olefinic alcohol esters for use as a fuel blend additive or as a precursor for longer-chain biofuels and biochemicals.

**Supplementary Information:**

The online version contains supplementary material available at 10.1186/s13068-023-02301-7.

## Background

Co-optimization of spark ignition (SI) engine and fuel performance is an outstanding challenge in the transportation sector with the potential for significant reductions in carbon intensity and economic expenditures [1, 2]. Advanced SI engines are characterized by a high compression ratio, downsizing, and downspeeding [1, 3]. However, improving advanced SI engine performance demands fuels with high resistance to autoignition (i.e., knocking) as typically defined by the research octane number (RON), motor octane number (MON), and octane sensitivity (OS; RON-MON). Robust databases (NREL Fuel Property Database; fuelsdb.nrel.gov) and quantitative structure-property models have been compiled to catalog and predict these fuel properties for a wide range of biofuel molecules. The resulting bioblendstocks have the potential to displace petrochemicals, improve advanced SI engine performance, and address the US Renewable Fuel Standards for a reduction in transportation sector emissions.

One of the main classes investigated, volatile aliphatic esters, are biologically ubiquitous, maintain unique fragrant properties, and serve as additives in cosmetics, food, solvents, coatings, and as biofuels [4, 5]. Ester bioproduction is attractive due to the relative ease with which alcohols and compatible thioesters may be metabolically paired with alcohol acyltransferases (AAT) for tailored ester production [5]. Pairing of an alcohol production pathway (e.g., ethanol, butanol, propanol, isopentanol), a native thioester (e.g., acetyl-CoA, butyryl-CoA, lactyl-CoA), and AATs has facilitated production of a veritable library of esters [6–8]. Common AATs include the *Saccharomyces cerevisiae* alcohol O-acetyltransferase 1 (ATF1), alcohol O-acetyltransferase 2 (ATF2), *Fragaria* x *ananassa* (strawberry) AAT (SAAT), and chloramphenicol acetyltransferase (CAT), with efficiencies contingent upon enzymatic specificity and precursor availability [9, 10].

Isoprenoids have also arisen as attractive biofuel candidates due to their favorable physicochemical properties, namely high energy densities, low viscosities, and high volumetric net heats of combustion [11]. Isoprenoids are biochemically composed of repeating olefinic C5 units derived from isopentenyl diphosphate (IPP) and dimethylallyl diphosphate (DMAPP), products of the metabolically independent but functionally similar mevalonate (MVA) and methylerythritol 4-phosphate (MEP) pathways. Conjugation of C5 units through various terpene synthases has yielded monoterpenes (limonene and 1,8-cineole) as well as sesquiterpenes (farnesene and bisabolene) as biodiesel or biojet fuel candidates with the hemiterpenoids isoprenol (3-methyl-3-buten-1-ol) and prenol (3-methyl-2-buten-1-ol) serving as potential gasoline additives [12]. Conventionally, isoprenol and prenol are produced through sequential dephosphorylation of IPP or DMAPP though growth and production are inhibited by toxic conjugation of excess IPP with ATP [13]. Recently, heterologous MVA pathway expression in *Escherichia coli* was engineered to bypass IPP inhibition through promiscuous activity of a modified diphosphomevalonate decarboxylase (PMD) [13–15]. Heterologous expression of this “IPP-bypass” yielded record titers of 3.7 g/L and 383.1 mg/L in *E. coli* and *S. cerevisiae* batch experiments, respectively [15, 16].

Isoprenol and prenol are unique bioblendstock candidates due to their strong synergistic RON and OS blending that enhances overall fuel performance [17, 18]. Prenol, for example, maintains a neat RON of 93.4 while a 20% prenol blend in gasoline can yield a RON of 99.3 [17]. This ability to boost the RON of a blend beyond the neat RON of the bioblendstock component is a phenomenon called hyperboosting and represents an extreme example of synergistic blending [17]. After first discovery in prenol blends, this effect was then observed in other olefinic isoprenoids including limonene, geraniol, and myrcene [19]. Recent works have expanded upon this observation, highlighting the proclivity of olefinic compounds to form stable intermediates often complemented by oxygen-containing functional groups [20]. Specifically, the electron delocalization of olefins facilitates H-atom abstraction and lends to favorable metastable radical formation, thereby improving knock-resistance and hence increasing fuel RON [21, 22]. Likewise, certain oxygenates including alcohols and some esters are enticing SI fuel additives due to the many metabolic pathways available for microbial bioproduction [20, 23].

Isoprenol and prenol are promising as bioblendstocks, but require host tolerance engineering or in situ product removal to overcome toxicity and achieve commercially viable titers when produced microbially [24]. While the introduction of an ester moiety could address these challenges and despite favorable titers of isoamyl acetate from isopentanol by ATF1 in *E. coli*, only incidental bioproduction of isoprenyl acetate titers have been demonstrated [25, 26]. Likewise, bioavailable unsaturated esters in particular have generally not be pursued as biofuel candidates due to antagonistic blending RON (bRON) and poor OS [18]. Esterification could simultaneously mitigate toxicity challenges and decrease downstream processing costs by reducing aqueous solubility and increasing volatility of the constituent alcohols, thereby improving the performance of in situ overlays for extractive fermentation or enabling facile recovery via distillation. This work investigates the coupling of isoprenoid biosynthesis with olefinic ester production, thereby marrying favorable oxygenate and olefinic properties to potentially yield excellent candidates with synergistic bRON and OS.

Here, we characterize the octane sensitivity of blends for a variety of olefinic esters in selected reformulated blendstocks for oxygenate blending (RBOBs), establishing blending octane sensitivity improves according to increased unsaturation and branching. We then demonstrate biological production of isoprenyl and prenyl acetate through heterologous expression of three AATs (ATF1, ATF2, and SAAT) and either the IPP-bypass or original MVA pathways, respectively. Low titer isoprenyl and prenyl lactate were generated by coupling their respective MVA pathway with heterologous SAAT expression and lactyl-CoA production. Finally, by further tuning acetate and acetyl-CoA flux in our ATF1 coupled IPP-bypass strain (JBEI-227475), we produced 28.0 g/L isoprenyl acetate under fed-batch conditions in minimal medium. This final titer represents 75.7% theoretical yield on glucose and establishes isoprenyl acetate not only as a promising advanced SI engine blend candidate, but as a potential precursor for isoprene and isoprene-derived products, including 1, 4-dimethylcyclooctane (DMCO), a drop-in replacement for high-performance Jet-A aviation fuel [27].

## Results

### Blend characterization of chemically synthesized esters

**Fig. 1:**
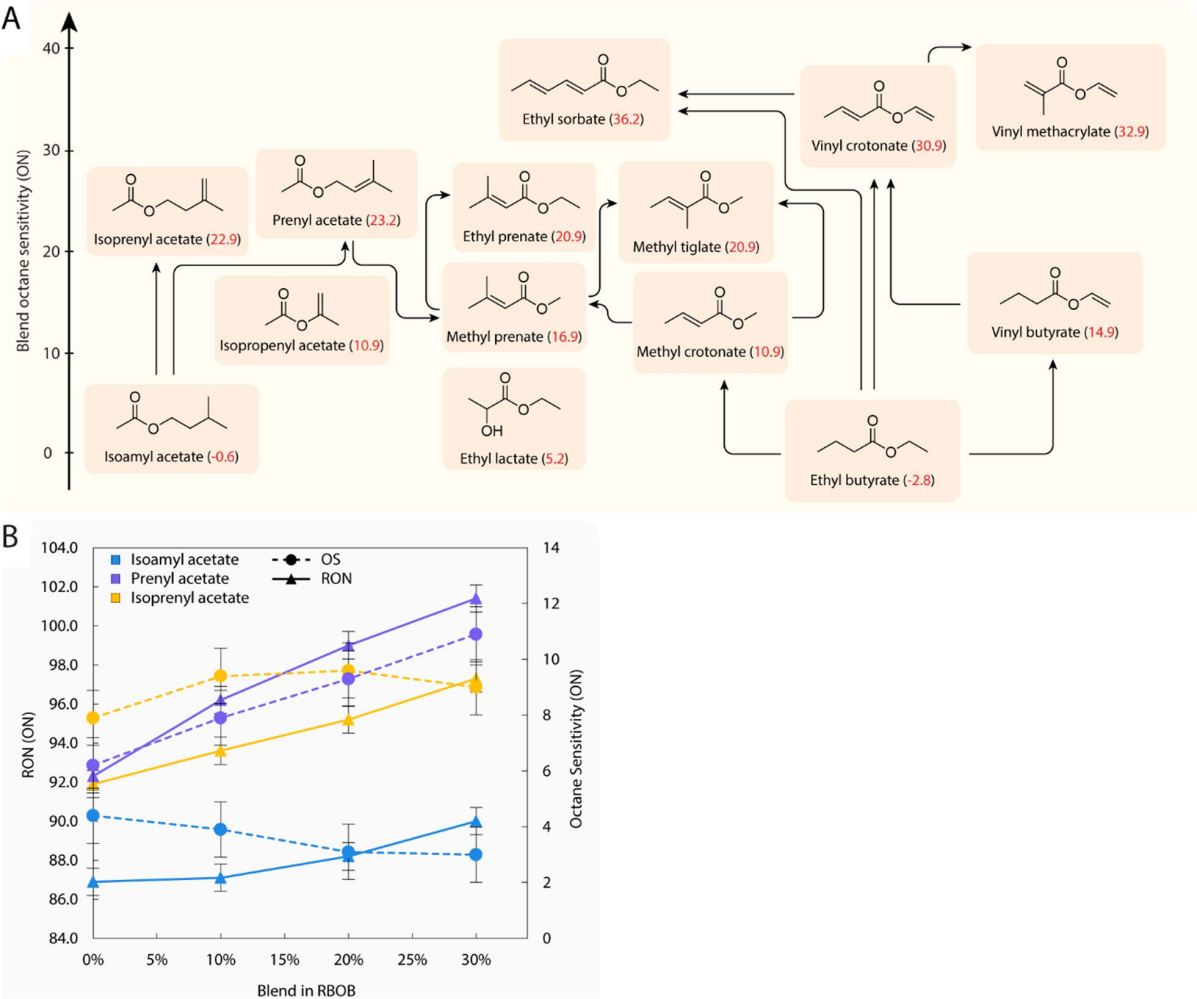
Fuel performance characterization of olefinic ester blends provided **A** 10% bOS by volume improvements (in red) that correlate with increased ester unsaturation and branching. Arrows loosely follow structural increases in unsaturation or chain length. **B** RON (solid line) and OS (dashed line) of prenyl acetate, isoprenyl acetate, and isoamyl acetate at 10%, 20%, and 30% blending by volume. Synergistic RON blending and strong OS boosting were exhibited for both prenyl and isoprenyl acetate in a 4-component surrogate gasoline fuel and RD587 (research gasoline fuel), respectively

Esters were blended in RBOBs for combustion analysis. The octane number (ON) is defined by a linear blending of *n*-heptane (*n*-C_7_H_16_) and *iso*-octane (*iso*-C_8_H_18_) with neat mixtures measuring 0 and 100 ON, respectively. A designated cooperative fuel research engine was run under distinctive conditions to ascertain RON and MON as outlined in ASTM D2699 and ASTM 2700, respectively, with the difference of between these values reported as the OS [28, 29]. From a measured RON or OS value of a blend, a metric called a blending octane number (bON) can be calculated, which quantifies the synergistic or antagonistic blending effect from the additive [30]. A bRON or bOS higher than the constitutive component neat RON or OS is synergistic blending, while a bRON or bOS lower than the neat components is antagonistic blending. To evaluate a full range of structure-function relationships, fourteen olefinic esters were synthesized and blended into a commercial RBOB. Figure 1A shows the bOS values and structural relationship with fuel properties in 10% RBOB (Fig. 1).

Broadly speaking, increases in ester unsaturation and branching, increased bOS (Fig. 1A). This is exemplified by dehydrogenation of ethyl butyrate (bOS = − 2.8) to vinyl butyrate (bOS = 14.9) and again to vinyl crotonate (bOS = 30.9) with increasing unsaturation sequentially increasing bOS further. The highest bOS measured was in ethyl sorbate with a bOS of 36.2. Of the olefinic esters investigated, isoprenyl and prenyl acetate were selected as the most promising candidates due to their high bRON (108.4 and 125.8 in 20% blends) and bOS (16.4 and 21.7 in 20% blends) values. Such synergistic blending was complemented by the fact they can be biologically produced via the metabolically straightforward conjugation of acetyl-CoA with isoprenol or prenol. Figure 1B investigates the RON and OS of prenyl acetate and isoprenyl acetate blends at different blend levels and demonstrates that both molecules display synergistic blending for both RON and OS. Likewise, both isomers clearly outperform their unsaturated analogue, isoamyl acetate. Differences in RON and OS between the two isomers stem from the primary vs. secondary allylic C–H bond position, likely resulting in better resonance stability and blend synergy for prenyl acetate [21]. MON, RON, OS, and corresponding blending data of isoamyl acetate, prenyl acetate, and isoprenyl acetate are listed in Additional file 1: Tables S1 and S2. Esters were blended in different but familiar base fuels owing to commercial availability. Details regarding fuel blending are available in the Materials and Methods section.

### Harnessing the MVA Pathway to generate olefinic esters

**Fig. 2:**
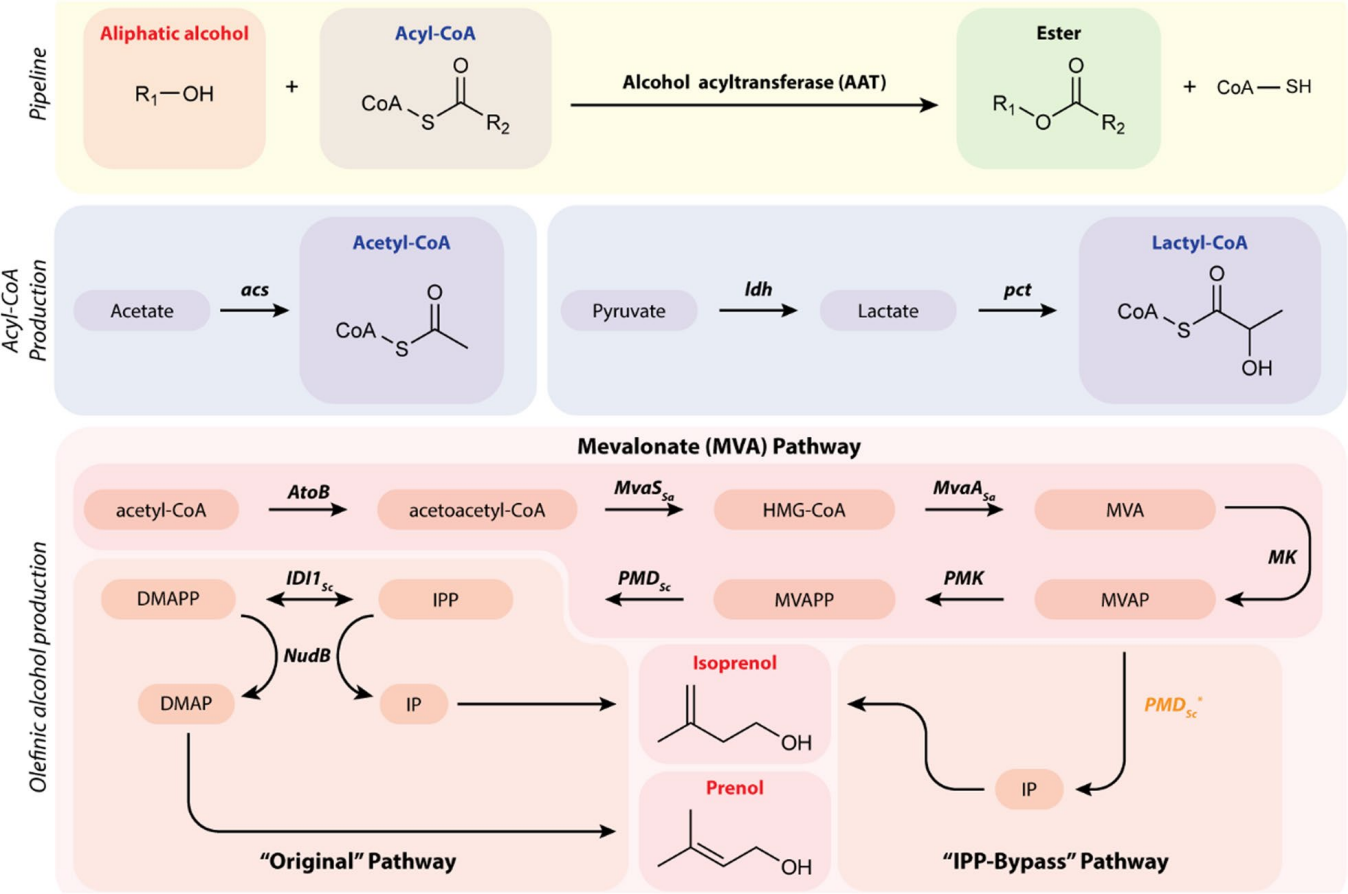
Biological production of isoprenoid esters may be accomplished through high production of isopentenol (isoprenol or prenol), overexpression of an AAT, and overproduction of intracellular acyl-CoA thioesters, in this case acetyl-CoA and lactyl-CoA

Microbial ester production pathways consist of three components: an alcohol, a thioester, and an alcohol acyltransferase (AAT) (Fig. 2) [7]. Traditionally, pathways have included a range of yeast and plant AATs with variable selectivity towards a library of common alcohols (ethanol, propanol, and butanol) as well as short chain length acyl-CoA thioesters; however, low specificity of AATs often lends to competing ester products contingent upon substrate availability [5].

Isoprenyl and prenyl acetate are novel ester candidates for production as fuel oxygenates derived from either the MEP or MVA pathways. The MVA pathway was selected for its demonstrated high titer production of C5 alcohols prenol and isoprenol [31, 32]. The canonical MVA pathway is a 6-enzyme cascade and, while nonnative to most bacteria, has been optimized through meticulous proteomics-informed tuning of selected heterologous genes in *E. coli* [33]. The pathway commences with a Claisen condensation of two acetyl-CoA molecules by a thiolase (AtoB; *E. coli*) to generate acetoacetyl-CoA, which is sequentially modified by 3-hydroxy-3-methylglutaryl-coenzyme A reductase (MvaA; *Staphylococcus aureus*) and a hydroxymethylglutaryl-CoA synthase (MvaS; *S. aureus*) to generate mevalonate. A mevalonate kinase (MK; *S. cerevisiae*) phosphorylates mevalonate to mevalonate monophosphate with further phosphorylation by mevalonate phosphate kinase (PMK; *S. cerevisiae*) to mevalonate diphosphate. Finally, a diphosphomevalonate decarboxylase (PMD; *S. cerevisiae*) generates IPP for downstream production of isoprenoids.

The delineation of the lower pathway into the “IPP-bypass” or “original” MVA pathways arises by inclusion of a promiscuous PMD that catalyzes formation of isopentenyl monophosphate (IP) directly from MVAP, simultaneously enabling conversion to isoprenol by an endogenous phosphatase and “bypassing” toxic IPP accumulation [13, 14, 32]. The bypass dramatically improves isoprenol production and strain growth. Conversely, the original MVA pathway includes an isopentenyl diphosphate isomerase (IDI) for interconversion of IPP to DMAPP. The nonselective dephosphorylation to their respective monophosphate via a phosphatase (NudB; *E. coli*), and conversion to their alcohol by an endogenous phosphatase(s) lends to an inherent mixture of isopentenol precursors.

Initial acyltransferase candidates included ATF1 and ATF2 from *S. cerevisiae*, which demonstrate selectivity towards primary alcohols and acetyl-CoA [34]. A third acyltransferase, SAAT from *F.* x *ananassa*, was also chosen for its proclivity for longer chain acyl-CoA thioesters. Selected acyltransferases, MVA pathways, and tertiary acyl-CoA generation plasmids were expressed in *E. coli* DH1.

### Production of acetate esters from MVA derived alcohols

Isoprenoid ester production was investigated through heterologous expression of ATF1, ATF2, or SAAT paired with either the IPP-bypass or original MVA pathways divided between two plasmids and cloned into *E. coli* DH1. Initial MVA pathway genes were expressed on a low copy plasmid harboring either AtoB, MvaA, and MvaS (IPP-bypass; JBEI-17844) or additionally with MK and PMK (Original MVA; JBEI-6829) under a strong IPTG-inducible promoter. A second, high copy plasmid harbored either PMD, MK, and selected AATs (IPP-bypass; JBEI-136483, JBEI-136484, or JBEI-231873) for isoprenyl acetate production or, following prenol optimization, NudB, IDI1, PMD, and ATF1 (Original MVA; JBEI-231880) for prenyl acetate production again under strong IPTG-inducible promoters.

Isoprenyl acetate was also investigated for potential toxicity towards *E. coli* DH1 under standard culturing conditions (Additional file 1: Figure S1). The addition of 2.5 g/L isoprenyl acetate at inoculation reduced OD_600_ by 57% over 24 h and higher concentrations resulted in complete growth inhibition. Moreover, only a fraction of isoprenyl acetate was detectable after 24 h owing to significant evaporative losses. Comparatively, cultures with a 20% oleyl alcohol organic overlay retained approximately 70% of initial isoprenyl acetate and *E. coli* continued to grow in concentrations up to 10 g/L. Considering these data, a 20% oleyl alcohol overlay was added upon IPTG induction to sequester ester products as well as to reduce evaporative losses and toxicity.

**Fig. 3:**
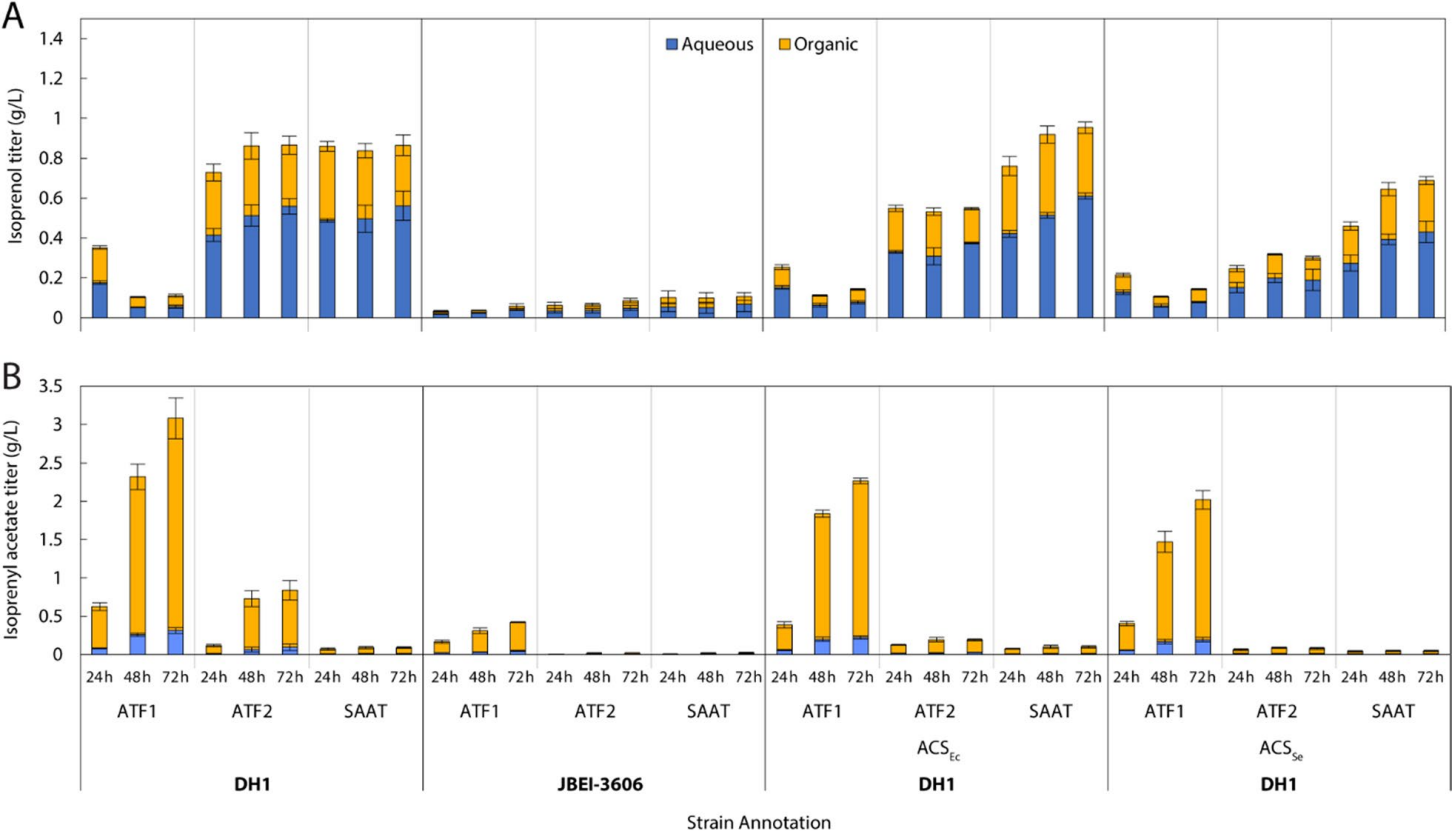
Flask production data of **A** isoprenol and **B** isoprenyl acetate in aqueous and organic phases from strains harboring the IPP-bypass pathway and specified AATs (ATF1, ATF2, or SAAT) in either *E. coli* DH1 or JBEI-3606 (*E. coli* DH1 Δ*poxB*, Δ*ackA*, Δ*pta*). Cultures were grown for 72 h in M9 medium (20 g/L glucose) with 5 g/L yeast extract and sampled every 24 h post-induction

Two acetyl-CoA synthetases (ACS) were also overexpressed to redirect acetate back into acetyl-CoA precursor. The first *acs* gene was cloned from *E. coli* chromosomal DNA (*acs*_*Ec*_) while another was cloned from *Salmonella enterica* (*acs*_Se_^L641P^). The latter *acs* harbors an L641P point mutation that disrupts the sirtuin-dependent acylation/deacylation system by preventing acetylation, effectively removing feedback inhibition for continual conversion of acetate into acetyl-CoA precursor [35–37]. Selected *acs* genes were cloned onto tertiary, low copy plasmids (pACS_Ec_/JBEI-231877 and pACS_Se_/JBEI-231876) and transformed into production strains. Lastly, bioproduction was demonstrated in both *E. coli* DH1 and a modified *E. coli* DH1 strain (JBEI-3606) characterized by complete knockouts of chromosomal pyruvate dehydrogenase (*poxB*), phosphate acetyltransferase (*pta*), and acetate kinase (*ackA*), which could reduce acetate formation and improve acetyl-CoA precursor flux through the MVA pathway (Fig. 3).

Pairing ATF1 with the IPP-bypass pathway (JBEI-137161) demonstrated exceptionally high olefinic ester production, attaining 3.08 ± 0.27 g/L over 72 h. Comparative production by ATF2 (JBEI-137162) and SAAT (JBEI-227453) were much lower, attaining maximum titers of 840 ± 135 mg/L and 94 ± 12 mg/L, respectively. Isoprenyl acetate also partitioned 90 ± 14% into the oleyl alcohol organic overlay, confirming improved partitioning when compared to direct isoprenol production [15]. Production in ATF2 and SAAT harboring strains was also accompanied by significant residual isoprenol titers of 866 ± 60 mg/L and 865 ± 90 mg/L, indicative of poor AAT specificity.

Typically, acetate pathway knockouts of *poxB* and *pta*-*ackA* (JBEI-3606) result in a near complete loss of acetate accumulation while improving available acetyl-CoA [15, 38]. However, expression of ATF1 in JBEI-3606 (JBEI-227474) resulted in 3.57 ± 1.05 g/L acetate accumulation (Additional file 1: Figure S2), a titer approximately fivefold higher than comparative strains harboring ATF2 (JBEI-231881; 0.67 ± 0.09 g/L) or SAAT (JBEI-231882; 0.68 ± 0.08 g/L). Acetate accumulation occurred in spite of acetate pathway KOs exclusively in JBEI-227474 stems from acetyl-CoA hydrolysis by ATF1 thioesterase promiscuity as previously observed in vitro [34]. Interestingly, the overexpression of *acs*_Se_^L641P^ or *acs*_*Ec*_ with ATF1 reduced isoprenyl acetate production by 34% and 26%, respectively, while disparately affecting titers in strains harboring ATF2 (JBEI-231883; 78% lower) or SAAT (JBEI-231884; 10% higher). Furthermore, acetate accumulation actually increased across all *acs* and AAT combinations examined (Additional file 1: Figure S2). The counterintuitive effect of *acs* expression on isoprenyl acetate accumulation emphasizes the nontrivial nature of acetate pathway rebalancing especially under batch conditions where glucose feeding and dissolved oxygen (DO) are unregulated. Nonetheless, pairing of the IPP-bypass with ATF1 produced an unprecedented titer of isoprenyl acetate that, in the context of its favorable blend properties, poses a significant opportunity for scalable oxygenate production. Full descriptions of strain genotypes and JBEI registry part number are available in Table 1.

**Table 1:** List of plasmids and strains used in this study

Plasmid or strain	JBEI Registry Part ID	Relevant genotype	Refs.
**Plasmid**			
pMTSA	JBEI-17081	pA5c-AtoB-HMGS_Sa-HMGR_Sa	[49]
IPPBy	JBEI-17844	ptrc99a-PMD_Sc_HKQ-MKmm	[15]
IPPBy-ATF1	JBEI-136483	ptrc99a-PMD_Sc_HKQ-MKmm-Atf1_Sc	This work
IPPBy-ATF2	JBEI-136484	ptrc99a-PMD_Sc_HKQ-MKmm-Atf2_Sc	This work
IPPBy-SAAT	JBEI-231873	ptrc99a-PMD_Sc_HKQ-MKmm-SAAT	This work
JBEI-6829	JBEI-6829	pA5c-AtoB-HMGS_Sa-HMGR_Sa-MKco-PMKco	[33]
NudBR10-PMD	JBEI-15848	pTrc99A-NudBR10-PMD_Sc	[39]
pDNC1	JBEI-231823	pTrc99A-IDI_EcR1-NudBR10-PMD_Sc	This work
pDNC2	JBEI-231824	pTrc99A-IDI_EcR2-NudBR10-PMD_Sc	This work
pDNC3	JBEI-231867	pTrc99A-NudBR10-IDI_EcR1-PMD_Sc	This work
pDNC4	JBEI-231868	pTrc99A-NudBR10-IDI_EcR2-PMD_Sc	This work
pDNC5	JBEI-231869	pTrc99A-IDI1_ScR1-NudBR10-PMD_Sc	This work
pDNC6	JBEI-231870	pTrc99A-IDI1_ScR2-NudBR10-PMD_Sc	This work
pDNC7	JBEI-231871	pTrc99A-NudBR10-IDI1_ScR1-PMD_Sc	This work
pDNC7-SAAT	JBEI-231879	pTrc99A-NudBR10-IDI1_ScR1-PMD_Sc-LacUV5-SAAT	This work
pDNC7-ATF1	JBEI-231880	pTrc99A-NudBR10-IDI1_ScR1-PMD_Sc-LacUV5-ATF1	This work
pDNC8	JBEI-231872	pTrc99A-NudBR10-IDI1_ScR2-PMD_Sc	This work
pDNC9	JBEI-231874	pBbE1k-ldhA_Ec-pct_Ap	This work
pDNC10	JBEI-231875	pBbE1k-ldh_Lm-pct_Ap	This work
pSAAT	JBEI-231763	pBbE1k-SAAT	This work
pACSse	JBEI-231876	pS5k-ACS_Se_L641P	This work
pACSec	JBEI-231877	pS5k-ACS_Ec	This work
**Strain**			
DH1	–	F^–^ λ^–^ endA1 recA1 relA1 gyrA96 thi-1 glnV44 hsdR17(r_K_^–^m_K_^–^)	Wild type
AceKO	JBEI-3606	DH1 Δpta, ΔackA, ΔpoxB	[50]
IPPBy	JBEI-137165	DH1 harboring JBEI-17081, JBEI-17844	[15]
DH1-IPPBy- ATF1	JBEI-137161	DH1 harboring JBEI-17081, JBEI-136483	This work
DH1-IPPBy-ATF2	JBEI-137162	DH1 harboring JBEI-17081, JBEI-136484	This work
DH1-IPPBy-SAAT	JBEI-227453	DH1 harboring JBEI-17081, pDNC33	This work
Original-MVA	JBEI-16126	DH1 harboring JBEI-6829, JPUB-004507; nudBRBS10	[39]
DNC1	JBEI-231891	DH1 harboring JBEI-6829, pDNC1	This work
DNC2	JBEI-231892	DH1 harboring JBEI-6829, pDNC2	This work
DNC3	JBEI-231893	DH1 harboring JBEI-6829, pDNC3	This work
DNC4	JBEI-231894	DH1 harboring JBEI-6829, pDNC4	This work
DNC5	JBEI-231895	DH1 harboring JBEI-6829, pDNC5	This work
DNC6	JBEI-231896	DH1 harboring JBEI-6829, pDNC6	This work
DNC7	JBEI-231897	DH1 harboring JBEI-6829, pDNC7	This work
DNC8	JBEI-231898	DH1 harboring JBEI-6829, pDNC8	This work
DNC7-ATF1	JBEI-231907	DH1 harboring JBEI-6829, pDNC7-ATF1	This work
DH1-IPPBy-ATF1-Acs_Ec	JBEI-227454	DH1 harboring JBEI-17081, IPPBy-ATF1, pACSec	This work
DH1-IPPBy-ATF1-Acs_Se	JBEI-227452	DH1 harboring JBEI-17081, IPPBy-ATF1, pACSse	This work
3606-IPPBy-ATF1	JBEI-227474	JBEI-3606 harboring JBEI-17081, IPPBy-ATF1	This work
3606-IPPBy-ATF1-Acs_Ec	JBEI-227475	JBEI-3606 harboring JBEI-17081, IPPBy-ATF1, pACSec	This work
3606-IPPBy-ATF1-Acs_Se	JBEI-227476	JBEI-3606 harboring JBEI-17081, IPPBy-ATF1, pACSse	This work
3606-IPPBy-ATF2	JBEI-231881	JBEI-3606 harboring JBEI-17081, IPPBy-ATF2	This work
3606-IPPBy-SAAT	JBEI-231882	JBEI-3606 harboring JBEI-17081, IPPBy-SAAT	This work
DH1-IPPBy-ATF2-Acs_Ec	JBEI-231883	DH1 harboring JBEI-17081, IPPBy-ATF2, pACSec	This work
DH1-IPPBy-ATF2-Acs_Se	JBEI-231885	DH1 harboring JBEI-17081, IPPBy-ATF2, pACSse	This work
DH1-IPPBy-SAAT-Acs_Ec	JBEI-231884	DH1 harboring JBEI-17081, IPPBy-SAAT, pACSec	This work
DH1-IPPBy-SAAT-Acs_Se	JBEI-231886	DH1 harboring JBEI-17081, IPPBy-SAAT, pACSse	This work
DH1-IPPBy-SAAT-ldha_Ec	JBEI-231903	DH1 harboring JBEI-17081, IPPBy-SAAT, pDNC9	This work
DH1-OriMVA-SAAT-ldha_Ec	JBEI-231905	DH1 harboring JBEI-6829, pDNC7-SAAT, pDNC9	This work
DH1-IPPBy-SAAT-ldh_Lm	JBEI-231904	DH1 harboring JBEI-17081, IPPBy-SAAT, pDNC10	This work
DH1-OriMVA-SAAT-ldh_Lm	JBEI-231905	DH1 harboring JBEI-6829, pDNC7-SAAT, pDNC10	This work
3606-IPPBy-SAAT-ldhA_Ec	JBEI-231899	JBEI-3606 harboring JBEI-17081, IPPBy-SAAT, pDNC9	This work
3606-OriMVA-SAAT-ldhA_Ec	JBEI-231901	JBEI-3606 harboring JBEI-6829, pDNC7-SAAT, pDNC9	This work
3606-IPPBy-SAAT-ldh_Lm	JBEI-231900	JBEI-3606 harboring JBEI-17081, IPPBy-SAAT, pDNC10	This work
3606-OriMVA-SAAT-ldh_Lm	JBEI-231902	JBEI-3606 harboring JBEI-6829, pDNC7-SAAT, pDNC10	This work
DH1-SAAT-ldhA_Ec	JBEI-231889	DH1 harboring pSAAT, pDNC9	This work
DH1-SAAT-ldh_Lm	JBEI-231890	DH1 harboring pSAAT, pDNC10	This work

**Fig. 4:**
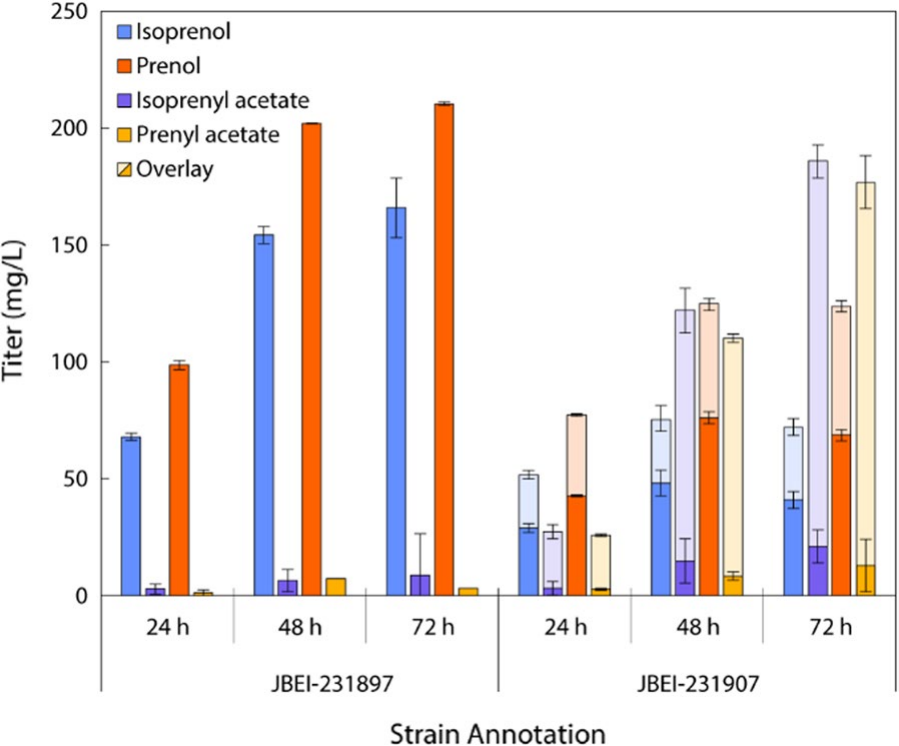
Flask production data from the original MVA pathway with optimized IDI1 expression (JBEI-231897) and further pairing with ATF1 (JBEI-231907). Ester production cultures included a 20% oleyl alcohol overlay upon induction, with partitioning depicted by opacity. Near equimolar concentrations of alcohols and esters were attained in each culture

Following successful isoprenyl acetate production, an analogous experiment was conducted by pairing ATF1 with the original MVA pathway to generate prenyl acetate. Previously, pairing the original MVA pathway with the Nudix hydrolase NudB and subsequent ribosome binding site (RBS) tuning yielded significant titer improvements of mixed isopentenols [39]. A series of strains overexpressing either an *S. cerevisiae* (IDI1) or *E. coli* (IDI_ec_) isomerase with predicted translation initiation rates equal to (RBS1) or twofold higher (RBS2) than NudB were also constructed to improve prenol production (Additional file 1: Figure S3) [40]. The best performing prenol production strain JBEI-231897, was then paired with ATF1 for prenyl acetate production (Fig. 4).

As expected, heterologous expression of the original MVA pathway and IDI1 resulted in a mixture of isoprenol and prenol with titers of 165.9 ± 12.7 mg/L and 210.4 ± 17.6 mg/L, respectively. Trace esters were detected likely due to CAT promiscuity with significant replicate variation stemming from a lack of overlay [26]. Pairing of an optimized prenol production strain (JBEI-231897) with ATF1 (JBEI-231907) and adding a 20% overlay resulted in approximately equimolar concentrations of isoprenyl and prenyl esters at titers of 185.7 ± 12.5 mg/L and 176.3 ± 16.0 mg/L, respectively. The approximate 20-fold reduction between IPP-bypass MVA isoprenyl acetate and original MVA prenyl acetate production arises due to routing flux through IPP and DMAPP. As a result, the original MVA pathway is not only enzymatically longer (MK, PMK, and IDI), but incurs isopentenyl diphosphate induced growth inhibition. Such growth inhibition is clear in comparing IPP-bypass coupled ATF1 growth (OD_600_ = 12.9 ± 0.4) against original MVA pathway coupled ATF1 growth (OD_600_ = 4.5 ± 0.7) over 72 h (Additional file 1: Figure S2, Figure S4). Generation of mixed esters and alcohols is also an inherent consequence of precursor isomerization. Double and triple fusions of IDI1, PMD, and NudB using variable length glycine–serine–glycine linkages were also explored due to previous indications of isopentenol titer improvements, though none outperformed JBEI-231897 (data not shown) [31, 39].

Nonetheless, JBEI-231897 demonstrated first prenyl acetate biosynthesis, validating a new biological production route for the olefinic ester with the most favorable bOS, bRON, and bMON values. Isomerically pure prenyl acetate production demands either isomerase optimization or an alternative metabolic strategy circumventing IDI altogether (e.g., isoprenol alcohol pathway) [41].

### Production of lactate esters from MVA derived alcohols

Given the favorable blend properties of the saturated ester ethyl lactate (bOS = 5.2), and the ease with which lactyl-CoA may be biologically generated, isoprenyl and prenyl lactate were investigated as further candidates. In conjunction with C5 alcohol production, a tertiary plasmid harboring a lactate dehydrogenase and a propionyl-CoA transferase combinedly convert pyruvate to a lactyl-CoA precursor (Fig. 2). SAAT was selected due to demonstrated activity towards longer chain thioesters. Initially, strain JBEI-231889 harboring an *ldhA*_*ec*_ from *E. coli*, *pct*_*Ap*_ from *Anaerotignum propionicum*, and SAAT from *F.* x *ananassa* was induced at high density (OD_600_ = 3.0) then supplemented with isoprenol to verify esterification feasibility. Chemically synthesized standards (Additional file 1: Figure S5, Figure S6) verified isoprenyl lactate accumulation with a marked excess of alcohol substrate (Additional file 1: Figure S7) but nonetheless demonstrating SAAT activity and lactyl-CoA production.

**Fig. 5:**
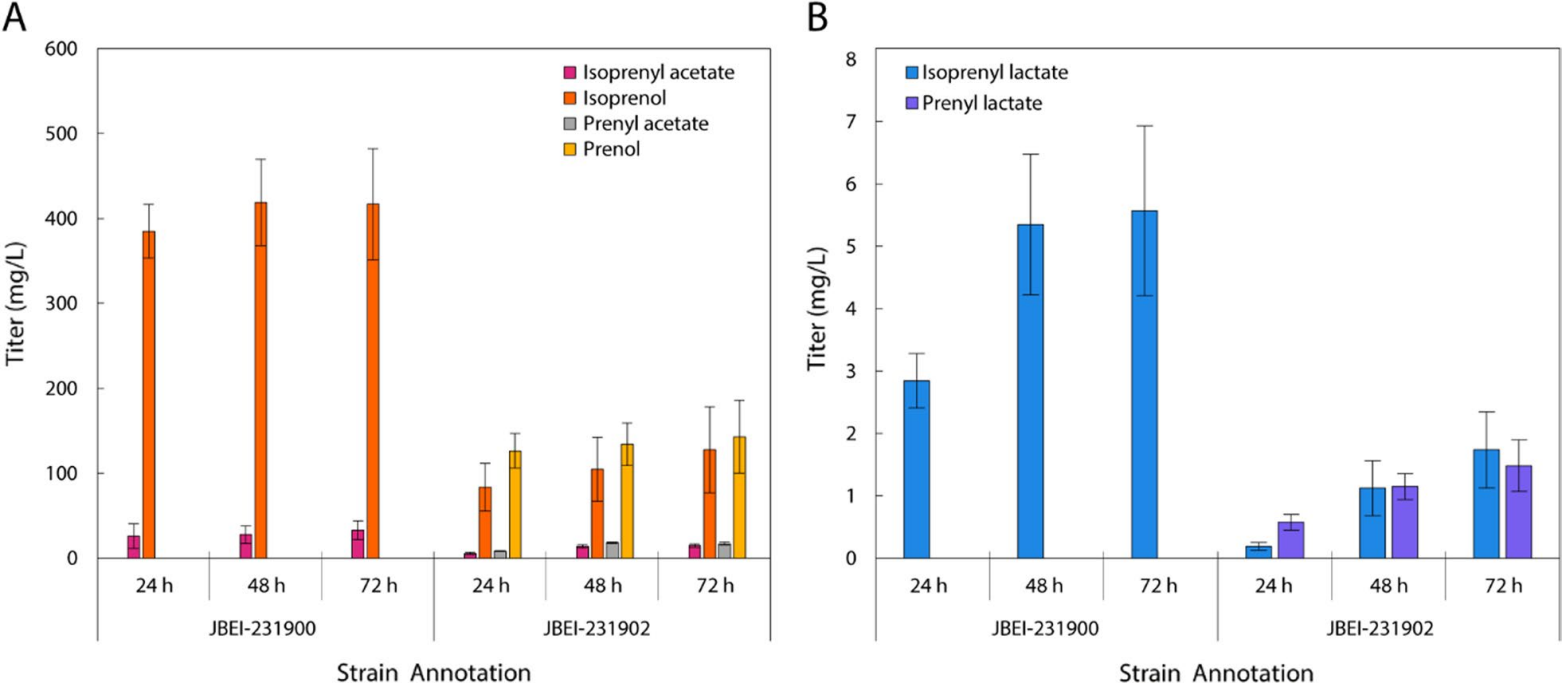
Flask production of **A** alcohols, acetate esters, and **B** lactate esters by strains harboring the lactyl-CoA production plasmid (*pct*_Ap_ and *ldh*_Lm_) along with SAAT and either the IPP-bypass pathway (JBEI-231900; 30 °C) or the original MVA pathway (JBEI-231902; 37 °C). Cultures were grown in M9-MOPS medium (20 g/L glucose) with 5 g/L yeast extract and without overlay

The generation of MVA-derived lactate esters juxtaposes two conflicting metabolic states; lactate accumulates under anaerobic conditions while the MVA pathway is inherently aerobic, relying on steady acetyl-CoA flux. To address this issue, a dehydrogenase from a microaerobic lactic acid forming bacteria, *Leuconostoc mesenteroides* subsp. *mesenteroides* (*ldh*_*Lm*_) was codon-optimized for expression in *E. coli*. The heterologous *ldh*_*Lm*_ demonstrated a 3.5-fold improvement in isoprenol conversion to isoprenyl lactate over the native *ldhA* (Additional file 1: Figure S7). Thus, *ldh*_Lm_ was paired with either the original or IPP-bypass MVA pathways for production at either 37 °C or 30 °C (Additional file 1: Figure S8), ultimately generating appreciable lactate ester accumulation (Fig. 5).

Pairing of the IPP-bypass pathway with SAAT and lactyl-CoA production (JBEI-231900) resulted in 5.57 ± 1.36 mg/L isoprenyl lactate. Likewise, use of the original MVA pathway (JBEI-231902) resulted in production of both lactate esters, attaining titers of 1.74 ± 0.21 mg/L isoprenyl lactate and 1.48 ± 0.41 mg/L prenyl lactate. Unsurprisingly, both pathways were accompanied by significant partner acetate ester titers and, as in the investigation of SAAT for isoprenyl acetate, significant isopentenols.

Ester titers in flask experiments were lower than preliminary culture tube experiments (10.0 ± 0.9 mg/L, 1.8 ± 0.6 mg/L; Additional file 1: Figure S8) owing to differences in culture conditions (i.e., aeration) and their influence on capricious strains harboring acetate pathway modifications. Glucose titer and optical density data indicate a cessation of growth after approximately 24 h (Additional file 1: Figure S9), which are consistent with the isoprenyl acetate flask experiments in the acetate pathway knockout strain. Nonetheless, significant residual lactate and alcohol necessitates further optimization of PCT or AAT for improving MVA-derived olefinic lactate ester titer.

### Optimization of isoprenyl acetate production in fed-batch fermentation

**Fig. 6:**
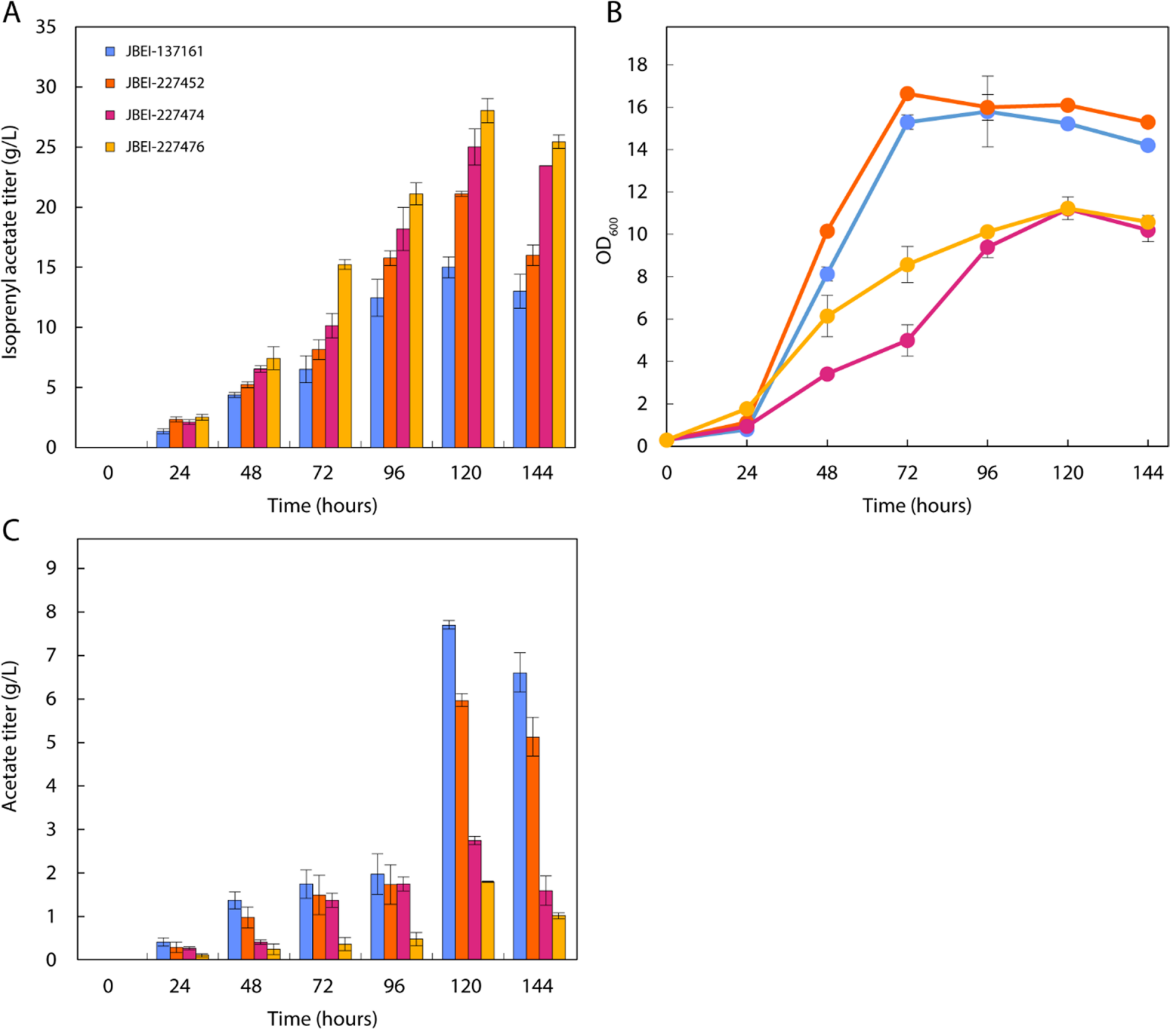
Fed-batch fermentation for isoprenyl acetate production. **A** Isoprenyl acetate accumulation over 144 h by selected production strains, achieving significant titer improvements with each optimization. **B** Biomass accumulation was significantly lower in the acetate pathway knockout strains JBEI-227452 and JBEI-227476 compared to their DH1 counterparts (JBEI-137161 and JBEI-227474), however **C** acetate pathway knockouts and acs_se_.^L641P^ provided significant, additive reductions in acetate titer and improvements in isoprenyl acetate titer (n = 3)

After favorable isoprenyl acetate production in flasks, JBEI-137161 and its acetate pathway variants were cultured in a 2-L bioreactor under fed-batch conditions. Acetate accumulation represents a loss of valuable acetyl-CoA substrate and eventually leads to growth inhibition at sufficient titers. By rebalancing acetate pathways as well as by controlling DO and glucose feed, we hypothesized that we could route carbon flux towards the MVA pathway, thereby improving isoprenyl acetate titer beyond what had been demonstrated in flask experiments. M9-MOPS minimal medium with 2% glucose was used during the batch fermentation while 80 g/L glucose and 5 g/L NH_4_Cl were continuously added at a constant rate over 144 h approximately 20 h after inoculation upon initial glucose depletion. All fed-batch fermentations were conducted using a 20% oleyl alcohol overlay to sequester isoprenyl acetate. Production of isoprenyl acetate from JBEI-137161 continuously increased during the fermentation, reaching a maximum titer of 15.0 ± 0.9 g/L (0.15 g/g_glucose_) at 120 h (Fig. 6).

Despite high titer isoprenyl acetate production, JBEI-137161 generated 7.82 ± 0.10 g/L acetate over 120 h, which resulted in significant precursor loss, growth inhibition and, consequently, limited ester production (Fig. 6B, C) [42]. Strains harboring acetate biosynthesis pathway knockouts (JBEI-227474) or *acs*_Se_^L641P^ overexpression (JBEI-227452) were then grown to ascertain whether acetate pathway rebalancing improved production under fermentation conditions. Fed-batch fermentation of JBEI-227452 resulted in 21.1 g/L isoprenyl accumulation over 120 h along with 6.06 ± 0.15 g/L acetate. JBEI-227474, however, produced 25.0 ± 1.5 g/L isoprenyl acetate with only 2.78 ± 0.10 g/L acetate accumulation. These strains demonstrated that the acetate pathway rebalancing strategy may prevent valuable substrate loss while improving conversion of glucose to ester product under fed-batch conditions. Finally, *acs*_*Se*_^*L641P*^ was paired with acetate pathway knockouts to improve acetyl-CoA precursor availability in JBEI-227476, attaining 28.0 g/L (0.28 g/g_glucose_) after 120 h with residual 1.80 ± 0.01 g/L acetate.

Acetate pathway rebalancing had a notably deleterious effect on biomass accumulation, but ultimately generated remarkable titers of isoprenyl acetate in JBEI-227452 and JBEI-227476. Maximum titers by all strains were achieved at 120 h owing to evaporative losses such that, under more stringent conditions, higher titers may still be possible. Nonetheless, assuming an MVA pathway efficiency of 0.78, an isoprenyl acetate titer of 28 g/L in minimal medium represents 75.7% theoretical yield on glucose (Additional file 1) [43].

## Discussion

Biologically derived olefinic oxygenates have tremendous potential for synergistic blending in advanced SI engines while reducing overall carbon intensity. Ester bioproduction has traditionally been hampered by poor AAT specificity and efficiency lending to low titer, rate, and yield. Likewise, bioproduction candidates have been limited to saturated esters with poor blend properties. By pairing MVA-derived alcohols with acetyl-CoA and lactyl-CoA thioesters, we demonstrated biosynthesis of novel isoprenyl and prenyl esters with favorable blend properties.

High titer isoprenyl and prenyl acetate production by ATF1 aligns with its demonstrated affinity for primary alcohols in general and towards isoamyl alcohol specifically [25, 26]. Here, ATF1 expression in isoprenoid alcohol producing *E. coli* resulted in highly efficient esterification of both prenol and isoprenol, indicative of high enzyme activity. However, significant acetate accumulation when coupling the IPP-bypass pathway with ATF1 in JBEI-227474 compared to either ATF2/SAAT confirmed ATF1 hydrolysis of acetyl-CoA, a phenomenon previously reported in vitro [34, 44].

**Fig. 7:**
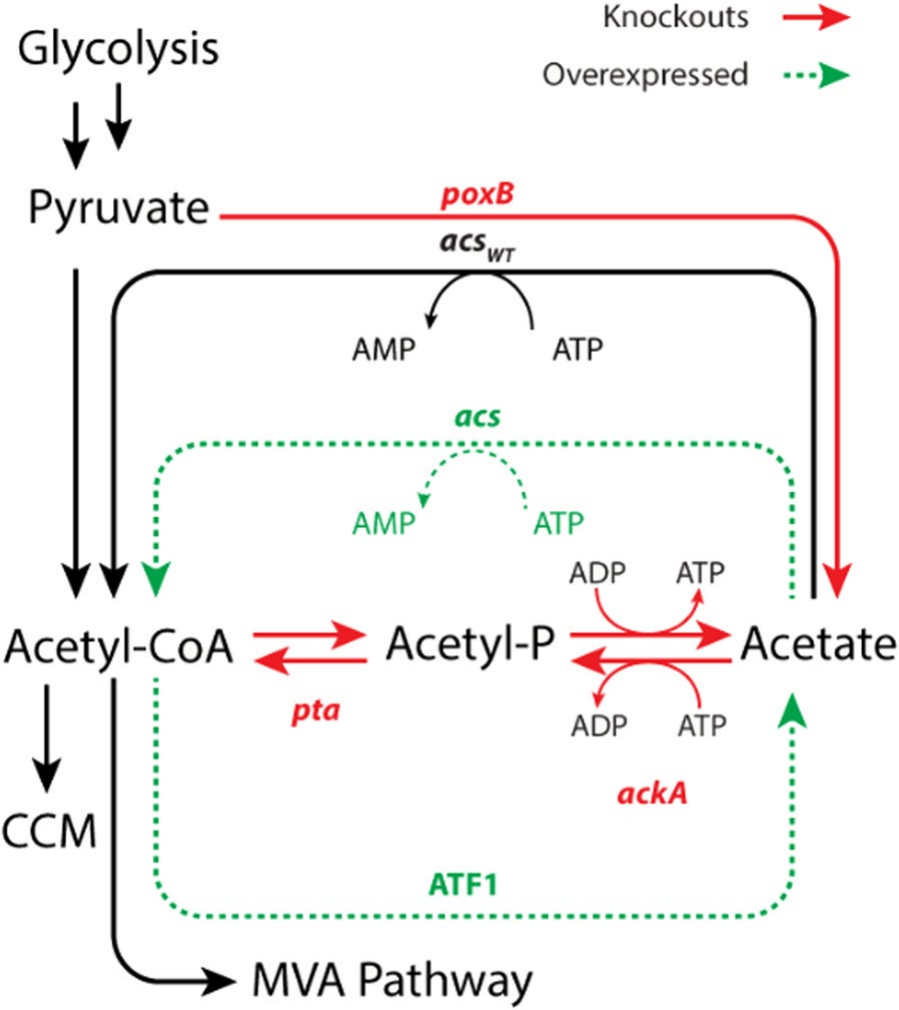
A simplified diagram of acetate flux rebalancing in selected fermenter strains accounting for hydrolysis of acetyl-CoA by ATF1, overexpression of *ACS*_*Se*_.^*L641P*^ or the native ACS (green), as well as acetate pathway knockouts (red) with respect to central carbon metabolism (CCM)

Acetyl-CoA availability is crucial for MVA pathway flux and especially pertinent for isoprenyl acetate production as the product is derived from three acetyl-CoA molecules [45]. Yet acetate pathway balancing is nontrivial, growth interdependent, and varies significantly with culture conditions [42, 46]. Interestingly, isoprenyl acetate yields by JBEI-137161 were identical under fed-batch and batch conditions (0.15 g/g_glucose_) but yields in acetate rebalanced strains differed dramatically. A plausible reason for these disparate results is that the synthetases overexpressed for acetyl-CoA regeneration are energetically burdensome compared to the native *pta*-*ackA* pathway. Pairing acetate rebalanced strains with ATF1 then generates a futile cycle in which ATF1 hydrolyzes acetyl-CoA to acetate while ACS catalyzes acetyl-CoA reconversion at high energetic cost (Fig. 7).

Furthermore, the lack of feedback inhibition in ACS_Se_^L641P^ exacerbates ATP demand. Collectively, ATP depletion and acetate accumulation may result in decreased growth and production. Fortunately, coupling acetate rebalancing with feed and aeration control under fed-batch conditions appears to rescue isoprenyl acetate titer by maintaining an aerobic environment and reducing overflow metabolism (Fig. 6). Aerobic conditions are essential for supplying ample ATP for MVA pathway flux as observed previously in the IPP-bypass pathway for isoprenol production [32]. Although these modifications proved successful in increasing fed-batch isoprenyl acetate production, optimization of acetate flux, energy balance, and protein expression could further improve overall titer, rate, and yield.

Precursor optimization is also crucial for improving prenyl acetate and lactate ester production. Production of prenyl acetate was comparatively low and accompanied by approximately equimolar isoprenyl acetate, an inherent characteristic of the IDI-mediated MVA pathway. Recently, an isoprenoid alcohol pathway (IAP) demonstrated growth-decoupled production of prenol, attaining titers over 2.0 g/L [41]. Although enzymatically longer than the IPP-bypass and demonstrated in rich medium, coupling the IAP with ATF1 could prove fruitful for pure prenyl acetate generation.

Provided the success of isoprenyl and prenyl acetate, we sought to expand our library of esters with regards to favorable blend candidate properties. Although not in our initial olefinic ester library, isoprenyl and prenyl lactate were selected due to the availability of alcohol precursor and favorable properties of the resultant ester product. Acyltransferases generate esters in which a β-carbon belongs to the parent alcohol. Computationally, esters in which the alkoxy group is in a β-position relative to the nearest chain end typically display more favorable fuel properties compared to their α-position analogues [21]. Of the esters characterized, most maintain an α-alkoxy group relative to the nearest chain end (Fig. 1A). While esterification with isoprenol/prenol would shift most selected ester α-alkoxy groups to the β-position, the resulting oxygenate chain lengths are prohibitive to gasoline blending. Lactate esters are a notable exception. Owing also to the favorable blend properties of ethyl lactate, isoprenyl and prenyl lactate were, therefore, selected as further candidates.

Although strains JBEI-231900 and JBEI-231902 successfully demonstrated lactate ester production, titers were low compared to their acetate analogues despite high lactate accumulation (Additional file 1: Figure S8). To our knowledge, *ldh*_*Lm*_ has not been expressed in prior ester pathways, though has proven efficacious for aerobic lactate ester production. Either lactate ester was accompanied by significant accumulation of its analogous acetate ester owing to SAAT specificity, though at much lower titers relative to ATF1. Low lactate ester production, therefore, stems from the poor affinity of SAAT towards acylating either alcohol, poor conversion of lactate to lactyl-CoA by PCT, or both [6]. Supposing the fuel properties characterization is favorable, it may be possible to optimize this pathway for improved specificity towards lactyl-CoA production/acylation. Nonetheless, the demonstrated affinity of SAAT for isopentenols and longer chain thioesters presents an opportunity to investigate other thioesters including propionyl-CoA, crotonyl-CoA, or 3-methylbutenoyl-CoA thereby further expanding olefinic ester production.

A major obstacle in ester production scaling is balancing titer against growth inhibition and evaporation. Indeed, significant isoprenyl acetate losses were observed in small-scale experiments (Additional file 1: Figure S1) due to its inherent volatility. Although the use of oleyl alcohol was effective, the scaling of isoprenyl acetate production demands more robust separation strategies that avoid such viscous nonpolar overlays altogether. Even at small scale, oleyl alcohol posed a significant challenge to liquid handling due to the difficulty of rapid organic phase separation for sampling and the high dilution factor necessary for chromatography. An opportunity for improving product separation is using a condenser in lieu of an organic overlay. Ester distillation wholly circumvents the necessity of an organic overlay while concentrating product for downstream applications.

Lastly, isoprenyl acetate may be deacetylated to isoprene and undergo iron-catalyzed [4 + 4]-cycloaddition with subsequent hydrogenation to 1,4-dimethylcyclooctane (DMCO) as a Jet-A substitute [47, 48]. While such production pathways have already been demonstrated with isoprene and isoprenol, isoprenyl acetate maintains distinctive safety, toxicity, and separation advantages. To maximize yield and carbon efficiency, deployment of isoprenoid esters as isoprene precursors would necessitate either recycling or valorization of the acetate leaving group. If this challenge can be overcome, bioproduction of isoprenyl acetate could enable jet fuel synthesis at lower carbon intensity than current petrochemical fuels.

## Conclusions

Advanced SI engines demand biofuels with higher resistance to autoignition to reduce cost, carbon emissions, and improve overall engine performance. Olefinic esters are excellent oxygenate candidates due to their propensity for synergistic blending, thereby increasing overall fuel bRON and bOS. Here, we demonstrated novel production of isoprenyl and prenyl acetate as well as isoprenyl and prenyl lactate in *E. coli*, each maintaining favorable characteristics as gasoline blendstocks. Isoprenyl acetate is both a candidate oxygenate and a potential precursor to isoprene and isoprene-derived sustainable aviation fuel. By harnessing the IPP-bypass MVA pathway, we successfully demonstrated high titer isoprenyl acetate production from glucose in minimal medium and, with further acetate pathway rebalancing, improved overall titer to 28.0 g/L at 75.7% of theoretical maximum yield.

## Materials and methods

### Construction of plasmids

All plasmids were constructed by pairing a PCR amplified DNA fragment with a vector harboring an origin and selection marker. All PCRs were performed using NEB Q5 polymerase according to the manufacturer’s instructions with primers listed in Additional file 1: Table S3. In the case of assemblies, PCRs were run with oligonucleotides (Integrated DNA Technologies) maintaining ~ 20 bp 5’ homology overhangs for scarless integration using NEBuilder HiFi Assembly Master Mix (NEB). Assemblages were then cloned into *E. coli* XL-1 Blue chemically competent cells for high efficiency plasmid replication. Plasmids harboring the desired sequence were subsequently isolated using a QIAprep Spin Miniprep Kit (Qiagen) and transformed into electrocompetent *E. coli* DH1 cells via electroporation (2500 V, 5 ms) in 0.2 cm gap cuvettes (Biorad).

To generate acetate esters, two alcohol O-acetyltransferase genes ATF1 [NCBI: NP_015022.3] and ATF2 [NCBI: NP_011693.1] were amplified directly from *S. cerevisiae* S288C chromosomal DNA and cloned 3’ of the mevalonate kinase in JBEI-17844 to generate JBEI-136483 and JBEI-136484, respectively, which harbors a mutagenized PMD enabling IPP-bypass. Another AAT [GenBank: AAG13130.1] from *F.* x *ananassa* denoted SAAT was codon optimized for *E. coli* and cloned 3’ of the mevalonate kinase of JBEI-17844.

Lactate esters were generated by cloning a codon optimized propionyl-CoA transferase (*pct*) from *A. propionicum* (formally *Clostridium propionicum*) [GenBank: WP066048121.1]*.* The selected *pct* was paired with a lactate dehydrogenase from *E. coli* (*ldhA*) or an *E. coli* codon optimized lactate dehydrogenase [GenBank: BAE80313.1] from *L. mesenteroides* subsp. *mesenteroides* (*ldh*_*Lm*_) to generate pDNC9 and pDNC10, respectively.

Plasmids constructed and used in this work are described in Table 1 as well as publicly available in the JBEI Registry (https://public-registry.jbei.org).

### Production culture conditions

Seed cultures were initially inoculated from single colonies into Luria–Bertani (LB) medium (10 g/L tryptone, 5 g/L yeast extract, and 10 g/L sodium chloride) for overnight growth at 37 °C on a rotary shaker at 200 rpm. Where applicable, antibiotics included carbenicillin (100 μg/mL), chloramphenicol (34 μg/mL), and kanamycin (50 μg/mL). The production medium was a variation of M9 minimal medium that included M9 salts (6.78 g/L Na_2_HPO_4_, 3 g/L KH_2_PO_4_, 1 g/L NH_4_Cl, and 0.5 g/L NaCl), 1 mg/L thiamine, 2 mM MgSO_4_, 10 nM FeSO_4_, 0.1 mM CaCl_2_, micronutrients (3*10^–8^ M (NH_4_)_6_Mo_7_O_24_, 4*10^–6^ M boric acid, 3*10^–7^ M CoCl_2_, 1.5*10^–7^ M CuSO_4_, 8*10^–7^ M MnCl_2_, and 1*10^–7^ M ZnSO_4_), as well as 75 mM 3-morpholinopropane-1-sulfonic acid (MOPS), 20 g/L glucose, appropriate antibiotics, and, if stated, 5 g/L yeast extract. Upon overnight growth in LB, seed cultures were inoculated into M9-MOPS medium with or without yeast extract for production or adaptation, respectively. Adaptation in M9-MOPS was found to be critical in reducing bacterial lag phase in medium without yeast extract.

Flask production cultures were inoculated at an optimal density (λ = 600 nm, 1 cm path length) of 0.05 in M9-MOPS using a Spectramax Plus spectrophotometer (Molecular Devices), then incubated at 37 °C with shaking at 200 rpm. Upon reaching an OD_600_ of 0.4 to 0.6, cultures were induced with 0.5 mM isopropyl *β*-D-1-thiogalactopyranoside (IPTG) and transferred to a shaker at 30 °C and 200 rpm. This experimental approach was used for both small-scale 5 mL culture tube and medium-scale 50 mL culture flask experiments. For acetate ester production, a 20% oleyl alcohol overlay was also added to sequester the relatively nonpolar products. Dodecane and hexadecane were also explored as overlay candidates, though oleyl alcohol maintained the best chromatogram clarity with respect to ester and alcohol cogeneration. Growth, metabolites, and production were determined daily using OD, high performance liquid chromatography (HPLC), and gas chromatography–flame ionization detector (GC–FID), respectively.

While acetate ester production was contingent on optimal MVA pathway conditions, lactate ester production required balancing lactate accumulation with C5 alcohol production. Titrating IPTG concentration (0.1 to 1.0) with induction optical density (0.2 to 1.0) had negligible improvements on 72-h lactate ester production. However, varying temperatures dramatically affected lactate ester production such that isoprenyl and prenyl lactate were cultured at 37 °C and 30 °C after induction, respectively (Additional file 1: Figure S9). Due to the dearth of production and expected lower volatility, no organic overlay was used in lactate ester cultures.

### Isoprenyl acetate production under fed-batch conditions

A 2 mL M9-MOPS seed culture was inoculated with 200 μL of a glycerol stock previously adapted for growth on minimal medium. The culture was grown for 12 h at 37 °C and 200 RPM. After 12 h, culture OD approached saturation (OD600 = 3.0) and a 100 μL aliquot was diluted 50-fold into fresh 5 mL M9-MOPS in triplicate, then grown for 24 h for medium adaptation. At saturation, the 5 mL cultures were organoleptically evaluated for the characteristic odor of isoprenyl acetate (fruity, green apple, and banana). If a strong odor was detected, 5 mL of the selected culture was transferred to 100 mL M9-MOPS medium in a baffled 1 L flask and grown at 37 °C and 200 RPM for approximately 8 h. After attaining an OD600 = 3.0, the entire 100 mL was then used to inoculate 900 mL fresh M9-MOPS medium in the fermentation vessel (initial OD600 = 0.3). Fed-batch fermentations were conducted in 2-L bioreactors (Sartorius BIOSTAT B plus). Upon reaching an OD600 between 0.6 and 0.8, culture temperature was reduced to 30 °C, and isoprenyl acetate production was induced with 0.5 mM IPTG. To prevent isoprenyl acetate evaporation, a 20% (v/v) oleyl alcohol overlay was added at the time of induction. Oleyl alcohol was also selected as the overlay for bioreactor experiments due to its favorable chemical properties, namely low foaming and high product solubility.

Setpoints for DO, temperature, and airflow were 30%, 30 ºC, and 1 vvm, respectively, for feedback control. Culture pH was maintained at 6.5 by supplementation with ammonia water (25%). A feeding solution (400 g/L glucose and 25 g/L NH_4_Cl) with a total feed volume of 100 mL delivered by a Watson-Marlow DU520 peristaltic pump after the initial glucose was depleted. The feed rate closely matched batch phase glucose consumption. The flow rate was increased every hour over a total of 8 h for exponential phase feeding and calculated following the Korz’s equation [15, 51].

$${m}_{s}(t)=(\mu /{y}_{(x/s)}+m)*{V}_{{t}_{F}}*{X}_{{t}_{F}}*{e}^{\mu (t-{t}_{F})}$$

Here, m_s_ is the mass flow of the substrate (g/hr) as a function of time (hr), µ is the specific growth rate of the strain (1/hr), y_x/s_ is the yield of biomass per unit of substrate (g/g_glucose_), and m assigns glucose consumed for cell maintenance (g/g_glucose_/hr). Lastly, V represents the cultivation volume (L) and X represents the biomass concentration at a given time (g_biomass_/L). After 8 h of exponential feeding, the feed rate remained constant and glucose concentration was measured consistently using a glucose meter (CVSHealth, USA) and HPLC to ensure that glucose concentration was less than 1 g/L.

As in the small-scale experiments, the organic phase and aqueous phase were first separated by centrifugation (for 10 min and at 5000 g), then the organic phase was analyzed via GC-FID to determine isoprenol and isoprenyl acetate concentration while the aqueous phase was used for measurements of optical density (OD_600_), acetate, and ethanol.

### Isoprenyl acetate toxicity assay

The relative differences in OD_600_ with and without organic overlay solvent provided strong evidence of isoprenyl acetate toxicity to *E. coli* DH1. The relative difference in ester vapor pressure and water solubility compared to their respective alcohols as well as the presence of a strong banana/green apple odor that aligned with the organoleptic characterization of isoprenyl acetate further suggested significant product losses due to evaporation. A simple assay was conducted to assess the toxicity of isoprenyl acetate on *E. coli* DH1 by titrating concentrations ranging between 0 g/L and 10 g/L, which is above the solubility limit of isoamyl acetate in water, into inoculated cultures in M9-MOPS as depicted as a fraction of remaining and total remaining isoprenyl acetate (Additional file 1: Figure S1).

### Quantification of alcohols and esters by gas chromatography

Upon sampling, organic and aqueous phases were separated by centrifugation at 13,000 g for 5 min. The use of an organic solvent like oleyl alcohol demanded 1:100 dilution in ethyl acetate prior to GC loading. For the isoprenyl acetate quantification from organic phase, 10 μL of oleyl alcohol was added to 990 μL ethyl acetate containing 1-butanol (30 mg/L) as an internal standard. Conversely, a fraction of isolated culture aqueous phase (250 μL) was mixed in a 1:1 ratio with ethyl acetate and vortexed for 10 min at 3000 rpm (Scientific industries INC, USA). Mixed phases were then centrifuged 13,000 g for 5 min with the organic phase diluted 1:5 in fresh ethyl acetate containing 1-butanol (30 mg/L) to a final volume of 1 mL. For high titer biofermenter production of isoprenyl acetate, most isoprenyl acetate partitioned into the overlay with only trace detectable isoprenyl acetate in the aqueous phase and overlay samples were further diluted due to high concentration. Ester concentrations were normalized across all scales according to the initial aqueous culture volume.

For the quantification of isoprenyl acetate, 1 μL of diluted samples was analyzed by GC-mass spectrometry (GCMS, Agilent, USA) and GC-FID (Focus GC-FID, Thermo Scientific, USA) equipped with a DB-WAX column (15 m, 0.32 mm inner diameter, 0.25 μm film thickness, Agilent, USA). The temperature program on the oven was set as follows: initiation at 50 ℃ and held for 1 min, a ramp of 15 ℃/min to 100 ºC and held 1 min, a ramp of 30 ℃/min to 230 ℃ and held at 230 ℃ for 1 min.

### Quantification of metabolites

Glucose, acetate, and, where relevant, lactate were quantified by HPLC (Agilent 1200 Series) using an Aminex HPX-87H column (Bio-Rad, Richmond, CA, USA). Aqueous culture samples were initially filtered through a 0.45 μM syringe, scrutinized for residual organic phase, then 10.0 μL was injected with a 0.005 M H_2_SO_4_ mobile phase. The flow rate was 0.6 mL/min with the column held at 65 ℃ and samples were analyzed using a relative index detector at 45 ℃.

### Chemical synthesis of analytical ester standards

A synthesis of isoprenyl acetate was conducted as described [52]. In brief, isoprenol (2.0 mL, 20 mM) was mixed with catalytic 4-dimethylaminopyridine (100 mg, 0.82 mM), and acetic anhydride (4.0 mL, 42.3 mM) at room temperature overnight. The reaction was quenched by the addition of DI H_2_O. The organic phase was extracted by addition of diethyl ether, then washed three times with saturated aqueous NaHCO_3_, separated, and dried over anhydrous MgSO_4_. The product was isolated by silica gel chromatography using a mixture of hexane and diethyl ether (9:1). Concentration under reduced pressure yielded a highly pure product as verified by GC-MS and ^1^H NMR (Bruker AVB-400) and confirmed with published data (https://webbook.nist.gov/).

Lactate esters were synthesized from 4-bromo-2-methylbut-1-ene (491 mg, 0.34 mM) (Combi-Blocks) or 1-chloro-3-methyl-3-butene (338 mg, 0.34 mM) (Sigma–Aldrich), which were mixed with D-lactic acid (347 mg, 0.31 mM) and NaCO_3_ (329 mg, 0.31 mM) in 50 mL dimethylformamide overnight. Upon completion, the reaction was quenched with DI H_2_O and extracted with ethyl acetate and hexane (3:7). The organic phase was washed three times with DI H_2_O. The product was isolated by silica gel chromatography and concentrated under reduced pressure. Characterization of the lactate esters was performed by GC-MS using the aforementioned method (Additional file 1: Figure S5) and ^1^H NMR (Bruker AVB-400, Additional file 1: Figure S6) for isoprenyl acetate, which aligned well with the literature [53]. NMR data and eventual GC-FID analysis of the prepared isoprenyl lactate standard indicated a slight prenyl lactate impurity from the halide reagent. As a result, the isoprenyl lactate product was used to quantify both lactate esters due to poor final yield of the prenyl lactate reaction. Analytical standards for isoprenol, prenol, and prenyl acetate were purchased from Sigma–Aldrich.

### Fuel property measurements

Where possible, the molecules shown in Fig. 1A were purchased at high purity from chemical vendors. Commercially unavailable esters were chemically synthesized at Sandia National Laboratories. All ester products were then sent to a commercial fuel testing lab, Intertek Inc., located in Benicia, California, United States where the biofuel candidates were volumetrically blended 10%, 20% and 30% blends in commercial hydrocarbon base fuels. Owing to commercial base gasoline availability, different base fuels were used across the duration of this study for blending, including 4-component surrogate (4CS), Sandia National Laboratories reformulated blendstock for oxygenate blending 4 (SNL RBOB4), and RD587. RON and MON were determined under standard operating conditions by ASTM D2699 and ASTM D2700, respectively, according to ISO/IEC 17,025 with accreditation by ANSI National Accreditation Board.

## Supplementary Information

**Additional file 1: Table S1.** RON, MON, and OS (RON-MON) of fuel blends and relevant oxygenates. **Table S2.** The blending RON, MON, and OS of specific oxygenates. **Table S3.** Primers used in this study. **Figure S1**. Isoprenyl acetate production and OD_600_ in M9-MOPS media over 24 hours. **Figure S2.** Acetate, glucose titer, and OD_600_ by strains harboring the IPP-bypass pathway with various AATs in *E. coli* DH1 or JBEI-3606. **Figure S3.** Prenol and isoprenol production by strains harboring different isomers (IDI/IDI1) and RBS strength. **Figure S4.** (A) Acetate, glucose titer, and OD_600_ of *E. coli *DH1 harboring the original MVA pathway with ATF1. **Figure S5.** GCMS for synthesized isoprenyl and prenyl lactate. **Figure S6.** ^1^H NMR of isoprenyl lactate with assignments. **Figure S7.** Ester production titrating isoprenol into high density cultures of *E. coli* DH1 harboring SAAT and either ldhA_Ec_ or ldh_Lm_. **Figure S8.** Small-scale production of lactate esters in DH1 and acetate pathway knockout strain JBEI-3606 at either 30 °C or 37 °C. **Figure S9.** (A) Acetate, lactate, glucose level, and OD_600_ of lactate ester strains. **Figure S10.** Production of isoprenol and isoprenyl acetate in strains harboring acetate pathway knockouts with *acs*_*Ec*_ or *acs*_*Se*_ in flask experiments.

## Data Availability

The dataset supporting the conclusions of this article is available in the JBEI’s Experiment Data Depot (https://edd.jbei.org/) and the strain information is available in the public version of the JBEI Registry (https://public-registry.jbei.org).

## Abbreviations

AAT: Alcohol acetyltransferase
ATF1: Alcohol O-acetyltransferase 1
ATF2: Alcohol O-acetyltransferase 2
bMON: Blending motor octane number
bOS: Blending octane sensitivity
bRON: Blending research octane number
CAT: Chloramphenicol acetyltransferase
CCM: Central carbon metabolism
DI: Deionized
DMAPP: Dimethylallyl diphosphate
DMCO: 1,4-Dimethylcyclooctane
GC–FID: Gas chromatography–flame ionized detector
GC–MS: Gas chromatography–mass spectrometry
HPLC: High-performance liquid chromatography
IDI: Isoprenyl diphosphate isomerase
IPP: Isopentenyl diphosphate
MEP: Methylerythritol-4-phosphate
MK: Mevalonate kinase
MON: Motor octane number
MVA: Mevalonate
OS: Octane sensitivity
PMD: Diphosphomevalonate decarboxylase
PMK: Mevalonate phosphate kinase
RBOB: Reformulated blendstock for oxygenate blending
RON: Research octane number
SAAT: Strawberry alcohol acyltransferase
