# Supplementary material for: Microbial production of high octane and high sensitivity olefinic ester biofuels
Source: Biotechnol Biofuels Bioprod. 2023 Apr 4;16:60. doi: 10.1186/s13068-023-02301-7 (PMC10071710; doi:10.1186/s13068-023-02301-7)
Supplement: Supplementary file 1 — Additional file 1: Table S1. RON, MON, and OS (RON-MON) of fuel blends and relevant oxygenates. Table S2. The blending RON, MON, and OS of specific oxygenates. Table S3. Primers used in this study. Figure S1. Isoprenyl acetate production and OD600 in M9-MOPS media over 24 hours. Figure S2. Acetate, glucose titer, and OD600 by strains harboring the IPP-bypass pathway with various AATs in E. coli DH1 or JBEI-3606. Figure S3. Prenol and isoprenol production by strains harboring different isomers (IDI/IDI1) and RBS strength. Figure S4. (A) Acetate, glucose titer, and OD600 of E. coli DH1 harboring the original MVA pathway with ATF1. Figure S5. GCMS for synthesized isoprenyl and prenyl lactate. Figure S6. 1H NMR of isoprenyl lactate with assignments. Figure S7. Ester production titrating isoprenol into high density cultures of E. coli DH1 harboring SAAT and either ldhAEc or ldhLm. Figure S8. Small-scale production of lactate esters in DH1 and acetate pathway knockout strain JBEI-3606 at either 30 °C or 37 °C. Figure S9. (A) Acetate, lactate, glucose level, and OD600 of lactate ester strains. Figure S10. Production of isoprenol and isoprenyl acetate in strains harboring acetate pathway knockouts with acsEc or acsSe in flask experiments. [file 13068_2023_2301_MOESM1_ESM.docx]

**Supplementary Information**

**Microbial production of high octane and high sensitivity olefinic ester biofuels**

David N. Carruthers^1,2^, Jinho Kim^1,2^, Daniel Mendez-Perez^1,2^, Eric Monroe^3^, Nick Myllenbeck^3^, Yuzhong Liu^1,2^, Ryan W. Davis^3^, Eric Sundstrom^1,4^, Taek Soon Lee^1,2,^*

^1^ Biological Systems and Engineering Division, Lawrence Berkeley National Laboratory, Berkeley, CA 94720, USA

^2^ Joint BioEnergy Institute, Emeryville, CA 94608, USA.

^3^ Sandia National Laboratories, Livermore, CA 94551, USA

^4^ Advanced Biofuels and Bioproducts Process Development Unit, Emeryville, CA 94608, USA

*Corresponding author, Joint BioEnergy Institute, 5885 Hollis Street, Emeryville, CA 94608, USA.

E-mail address: tslee@lbl.gov (T. S. Lee). Tel: +1-510-495-2470. Fax: +1-510-495-2629.

**Table S1.** RON, MON, and OS (RON-MON) of fuel blends and relevant oxygenates, namely isoamyl acetate, prenyl acetate, and isoprenyl acetate. Different oxygenates were tested in slightly different base fuels, including 4-component surrogate (4CS), Sandia National Laboratories reformulated blendstock for oxygenate blending 4 (SNL RBOB4), and RD587 is another research gasoline designation. Isoamyl acetate data collected from https://fuelsdb.nrel.gov^1^.

|  |  | | **RON** | | | | | **MON** | | | | | **OS** | | | | |
| --- | --- | --- | --- | --- | --- | --- | --- | --- | --- | --- | --- | --- | --- | --- | --- | --- | --- |
| **Compound (%)** | | **Base Fuel** | *0%* | *10%* | *20%* | *30%* | *Neat* | *0%* | *10%* | *20%* | *30%* | *Neat* | *0%* | *10%* | *20%* | *30%* | *Neat* |
| Isoamyl acetate^1^ | | SNL RBOB4 | 86.9 | 87.1 | 88.2 | 90 | 98.8 | 82.5 | 83.2 | 85.1 | 87 | 86.8 | 4.4 | 3.9 | 3.1 | 3.0 | 12 |
| Prenyl acetate | | 4CS | 92.3 | 96.2 | 99 | 101.4 | 104.8 | 86.1 | 88.3 | 89.7 | 90.5 | 94.4 | 6.2 | 7.9 | 9.3 | 10.9 | 10.4 |
| Isoprenyl acetate | | RD587 | 91.9 | 93.6 | 95.2 | 97.3 | 103.3 | 84 | 84.2 | 85.6 | 88.3 | 94 | 7.9 | 9.4 | 9.6 | 9.0 | 9.3 |

**Table S2.** The blending RON, MON, and OS of specific oxygenates. The bRON and bMON are calculated as the RON/MON of a given blend minus the product of base fuel blend fraction and base fuel RON/MON, then divided by the oxygenate blend fraction (e.g., a 10% blend calculation is (RON_oxygenate_ - (0.9) * RON_Base fuel_) / (0.1)). A linear blend would provide a bRON or bMON equal to that of the base fuel and a bOS of 0. Isoamyl acetate data collected from https://fuelsdb.nrel.gov^1^.

|  | |  | **bRON** | | | **bMON** | | | **bOS** | | |
| --- | --- | --- | --- | --- | --- | --- | --- | --- | --- | --- | --- |
| **Compound (%)** | **Base Fuel** | | *10%* | *20%* | *30%* | *10%* | *20%* | *30%* | *10%* | *20%* | *30%* |
| Isoamyl acetate^1^ | SNL RBOB4 | | 88.9 | 93.4 | 97.2 | 89.5 | 95.5 | 97.5 | -0.6 | -2.1 | -0.3 |
| Prenyl acetate | 4CS | | 131.3 | 125.8 | 122.6 | 108.1 | 104.1 | 100.8 | 23.2 | 21.7 | 21.9 |
| Isoprenyl acetate | RD587 | | 108.9 | 108.4 | 109.9 | 86 | 92 | 98.3 | 22.9 | 16.4 | 11.6 |

**Table S3.** Primers used in this study

| **Primer Name** | **Sequence (5’ → 3’)** |
| --- | --- |
| ATF1_F | ggataggaggattacactatgaatgaaatcgatgagaaaa |
| ATF1_R | catccgccaaaacagccaagctaagggcctaaaaggagag |
| IPPBy-ATF1_R | agtgtaatcctcctatccttagtctactttcagaccctgct |
| IPPBy-ATF1_F | agctctccttttaggcccttagcttggctgttttggcgga |
| ATF2_F | ggataggaggattacactatggaagatatagaaggata |
| ATF2_R | catccgccaaaacagccaagttaaagcgacgcaaattcgc |
| IPPBy-ATF2_R | agtgtaatcctcctatccttagtctactttcagaccct |
| IPPBy-ATF2_F | gcgaatttgcgtcgctttaacttggctgttttggcggatg |
| SAAT_F | acaatttcaggatctggatcttctggtgggtctctgt |
| SAAT_R | tcgaaaaatcggacattacctcctagttacttaaatcaacgtttt |
| IPPBy-SAAT_F | tctccttttaggcccttagcttggctgttttggcg |
| IPPBy-SAAT_R | ttctcatcgatttcattcatagtgtaatcctcctatcctt |
| Acs_Se_F | tttaagaaggagatatacatatgagccaaacacataaaca |
| Acs_Se_R | ttactcgagtttggatccttatgacggcatcgc |
| Acs_Ec_F | tttaagaaggagatatacatatgagccaaattcacaaaca |
| Acs_Ec_R | ttactcgagtttggatccttacgatggcatcgcgatag |
| pAcs_F | cgatgccgtcataaggatccaaactcgagtaa |
| pAcs_R | tgtttatgtgtttggctcatatgtatatctccttcttaaaagat |
| IDI_RB1_F | cagggatactaacttatagggatagctttatgcaaacggaacacgtcat |
| IDI_NudB_R | ccttcttaaaagatcttttgaattcccatggtttatttaagctgggtaaatgcag |
| IDI_RB2_F | agattctacgatttcttcccgggggtttagtatgcaaacggaacacgtcat |
| IDI_PMD_R | cctcctagatccgaactcgagtttggatccttatttaagctgggtaaatg |
| pDNC_IDI_NudB_F | gccagaaaacgattatctgcatttacccagcttaaataaaccatgggaattcaaaagatc |
| pDNC_RB1_R | aaagctatccctataagttagtatccctgctgtttcctgtgtgaaattgt |
| pDNC_RB2_R | actaaacccccgggaagaaatcgtagaatctctgtttcctgtgtgaaatt |
| pDNC_IDI_PMD_F | cgattatctgcatttacccagcttaaataaggatccaaactcgagttcgg |
| pDNC_NudB_RB1_R | aaagctatccctataagttagtatccctgtcaggcagcgttaattacaa |
| pDNC_NudB_RB2_R | actaaacccccgggaagaaatcgtagaatcttcaggcagcgttaattacaa |
| IDI1_RB1_F | cagggatactaacttatagggatagctttatgactgccgacaacaatag |
| IDI1_NudB_R | ctccttcttaaaagatcttttgaattcccatggtttatagcattctatgaatttgcc |
| IDI1_RB2_F | agattctacgatttcttcccgggggtttagtatgactgccgacaacaata |
| IDI1_PMD_R | cctcctagatccgaactcgagtttggatccttatagcattctatgaattt |
| pDNC_IDI1_NudB_F | gtggaaaatgacaggcaaattcatagaatgctataaaccatgggaattcaaaagatc |
| pDNC_IDI1_PMD_F | aatgacaggcaaattcatagaatgctataaggatccaaactcgagttcgg |
| SAAT2_F | acaatttcaggatctggatcttctggtgggtctctgt |
| SAAT2_R | tccttactcgagtttggatccttaaatcaacgttttggga |
| pSAAT_F | tcccaaaacgttgatttaaggatccaaactcgagtaa |
| pSAAT_R | acagagacccaccagaagatccagatcctgaaattgttat |
| pDNC7-ATF1_F | ttttaggcccttagctgcaggcatgcaa |
| pDNC7-ATF1_R | atcaccggcgccacaatttattcctttggtaga |
| LacUV5_F | tctaccaaaggaataaattgtggcgccggtgat |
| LacUV5_R | tagtgtaatcctcctatccgatcttttgaattctgaaatt |
| ATF1-Ins_F | aatttcagaattcaaaagatcggataggaggattacacta |
| ATF1-Ins_R | ttgcatgcctgcagctaagggcctaaaa |
| pDNC7-SAAT_F | tcccaaaacgttgatttaacttggctgttttggcg |
| pDNC7-SAAT_R | cgacacttcaatcttctccatagtgtaatcctcctatcc |
| SAAT3_F | ggataggaggattacactatggagaagattgaagtgtcg |
| SAAT3_R | cgccaaaacagccaagttaaatcaacgttttggga |
| pDNC9_ldhA_F | gagatgaagagctaactggatccatgtgattcaacatcac |
| pDNC9_ldhA_R | tttgatgcctggagatccttactcgagttttaaaccagtt |
| pDNC9_pct_F | tttaagaaggagatatacatatgcgcaaggtgcctatcat |
| pDNC9_pct_R | gtgatgttgaatcacatggatccagttagctcttcatctc |
| pDNC9_F | aactggtttaaaactcgagtaaggatctccaggcatcaaa |
| pDNC9_R | atgataggcaccttgcgcatatgtatatctccttcttaaa |
| pDNC10_Ldh_lm_F | aaagagatgaagagctaactgaattcgccggaccc |
| pDNC10_ldh_lm_R | ttactcgagtttggatccttaatactcgacggcgatg |
| pDNC10_pct_F | atcgccgtcgagtattaagaattctcgataaacgagat |
| pDNC10_pct_R | atccttactcgagtttggatccagttagctcttcatctcttttaatcccat |
| pDNC10_F | agagatgaagagctaactggatccaaactcgagtaaggat |
| pDNC10_R | ccataggcaaaaatcttcatgaaaagcctctctatg |

**
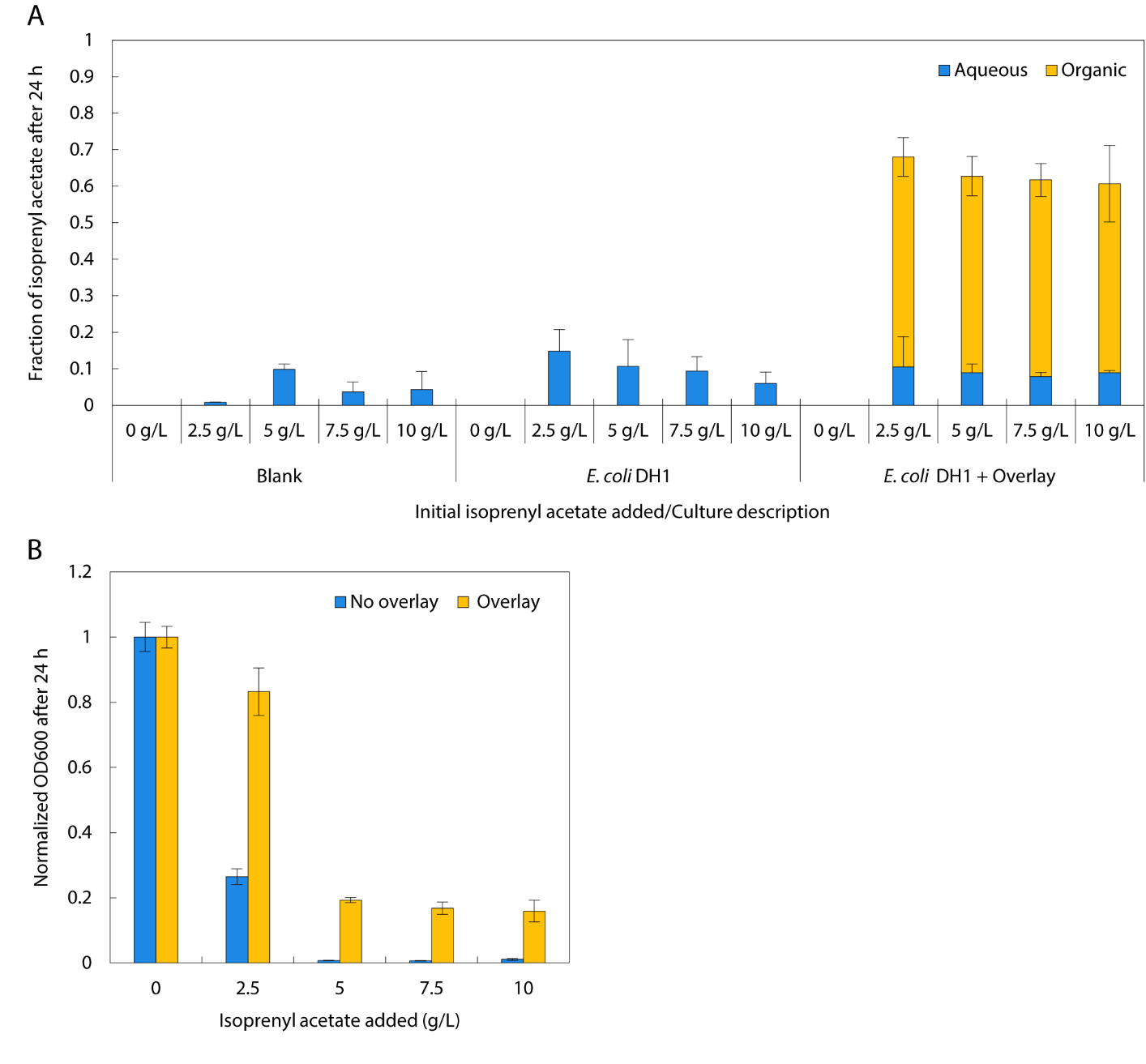
**

**Figure S1**. Isoprenyl acetate (A) and *E. coli* DH1 OD­_600_ (B) in M9-MOPS media over 24 hours normalized to 0 g/L isoprenyl acetate growth. Use of a 20% oleyl alcohol overlay enabled 75% retention of isoprenyl acetate, though significant growth inhibition was observed at high concentrations.

**
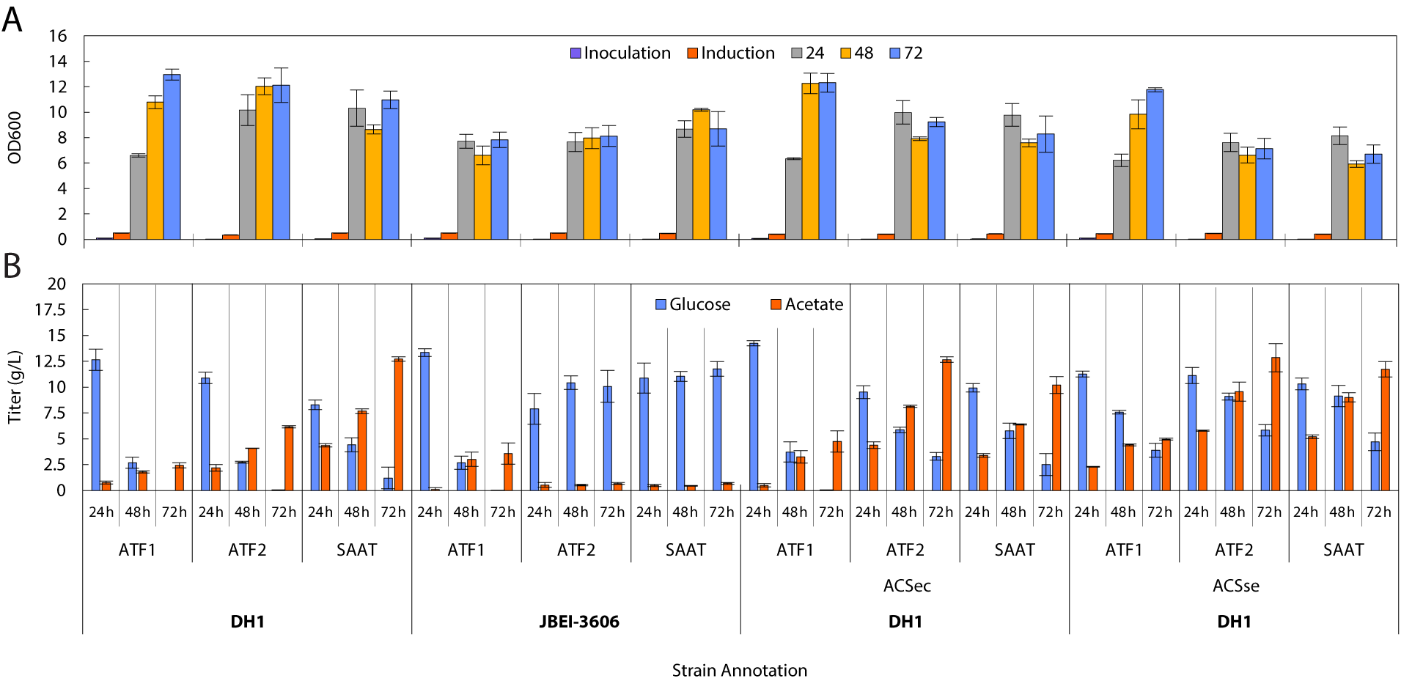
**

**Figure S2.** (A) Acetate and glucose titer by strains harboring the IPP-bypass pathway with various AATs in either *E. coli* DH1 or JBEI-3606 (*E. coli* DH1 Δ*poxB*, Δ*ackA*, Δ*pta*) and (B) OD_600_ growth data. Cultures were grown in M9 (20 g/L glucose) with 5 g/L yeast extract. Full strain descriptions are available in Table 1.


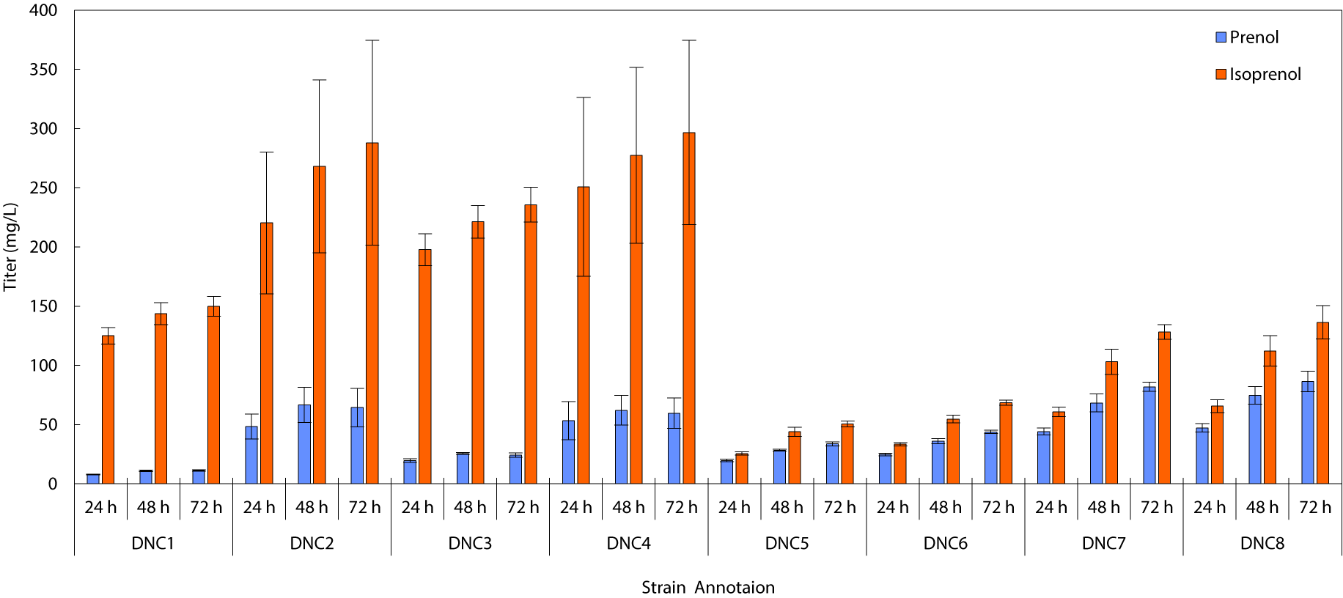


**Figure S3.** Prenol and isoprenol production by strains harboring different isomers (IDI/IDI1) and RBS strength (RBS1 and RB2) for prenol production (pDNC1-8). RBS1 maintains a predicted translation initiation rate approximately equivalent to NudB while RBS2 is 2-fold higher [[1]](https://sciwheel.com/work/citation?ids=348364&pre=&suf=&sa=0&dbf=0). Strains are described in detail in Table 1.

**
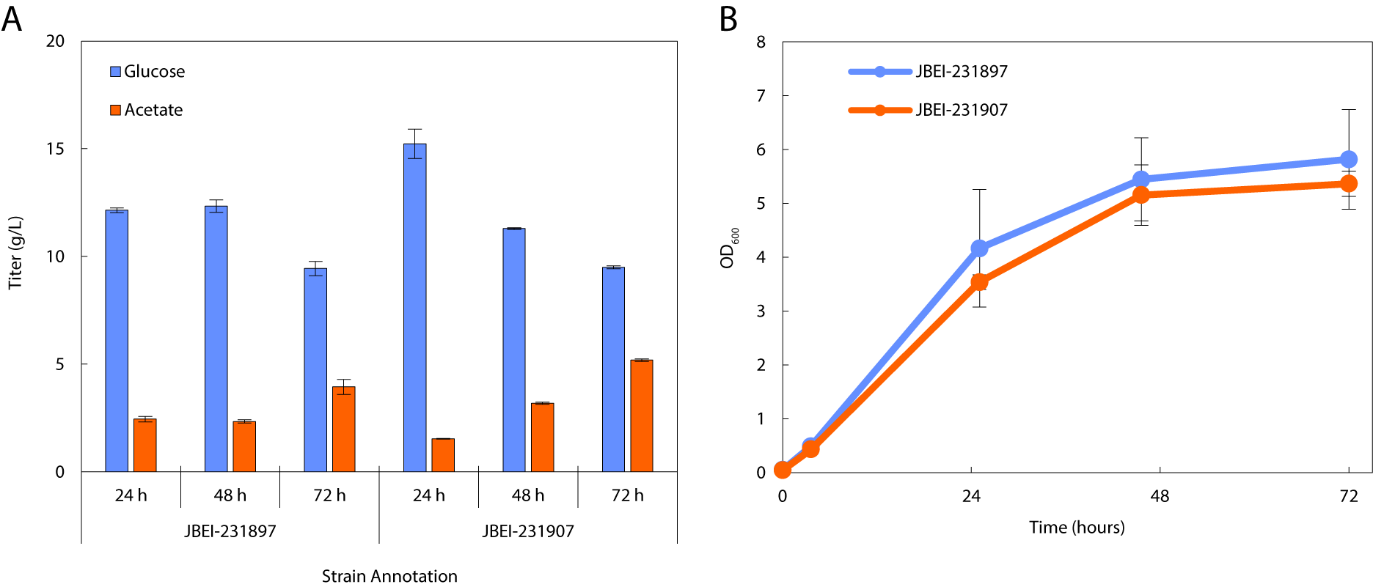
**

**Figure S4.** (A) Acetate and glucose titer by *E. coli* DH1 harboring the original MVA pathway with ATF1 and (B) OD_600_ growth data. Cultures were grown in M9 Medium (20 g/L glucose) with 5 g/L yeast extract.

**
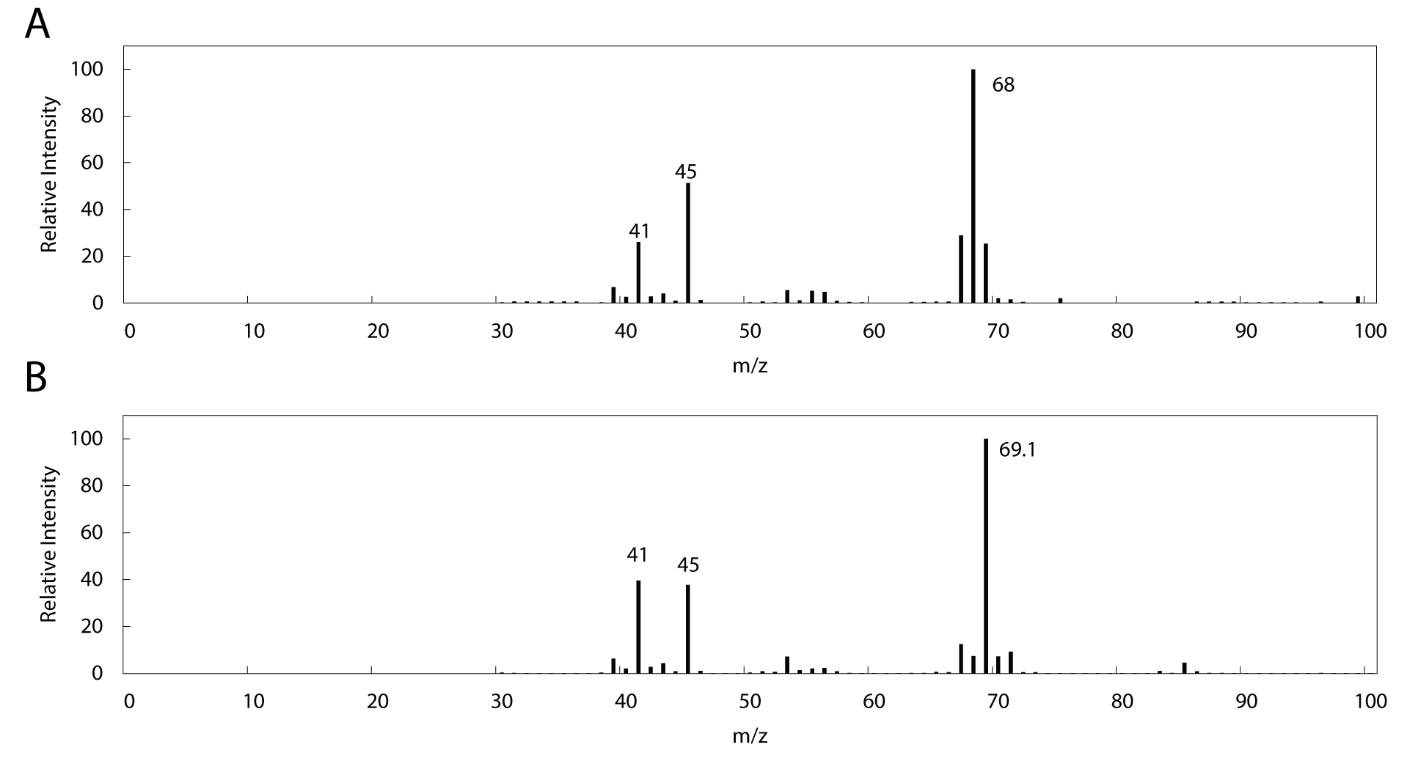
Figure S5**: GCMS m/z plot for synthesized (A) isoprenyl and (B) prenyl lactate. High abundance fragment ions are labeled


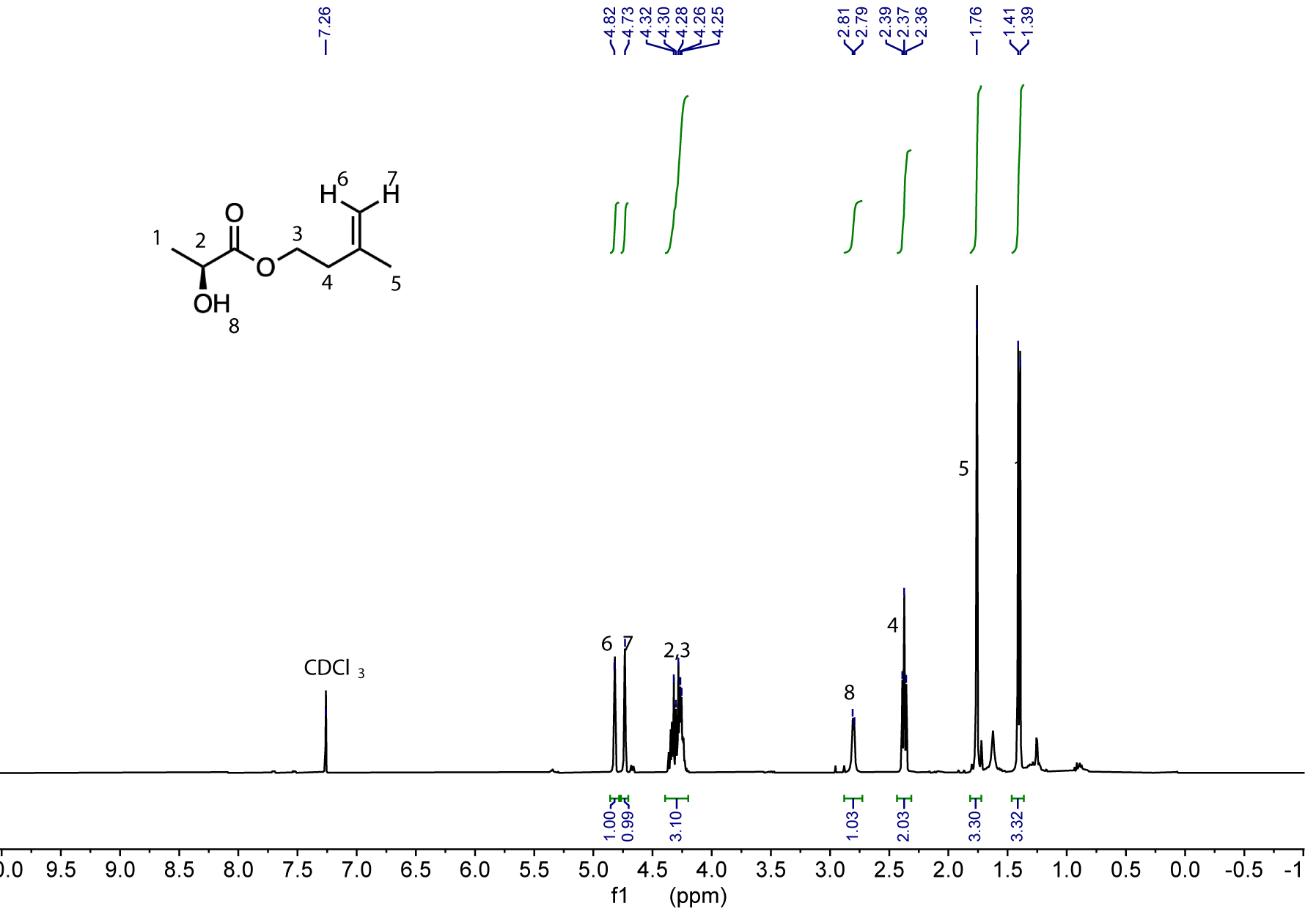


**Figure S6.** ^1^H NMR of isoprenyl lactate with assignments

**
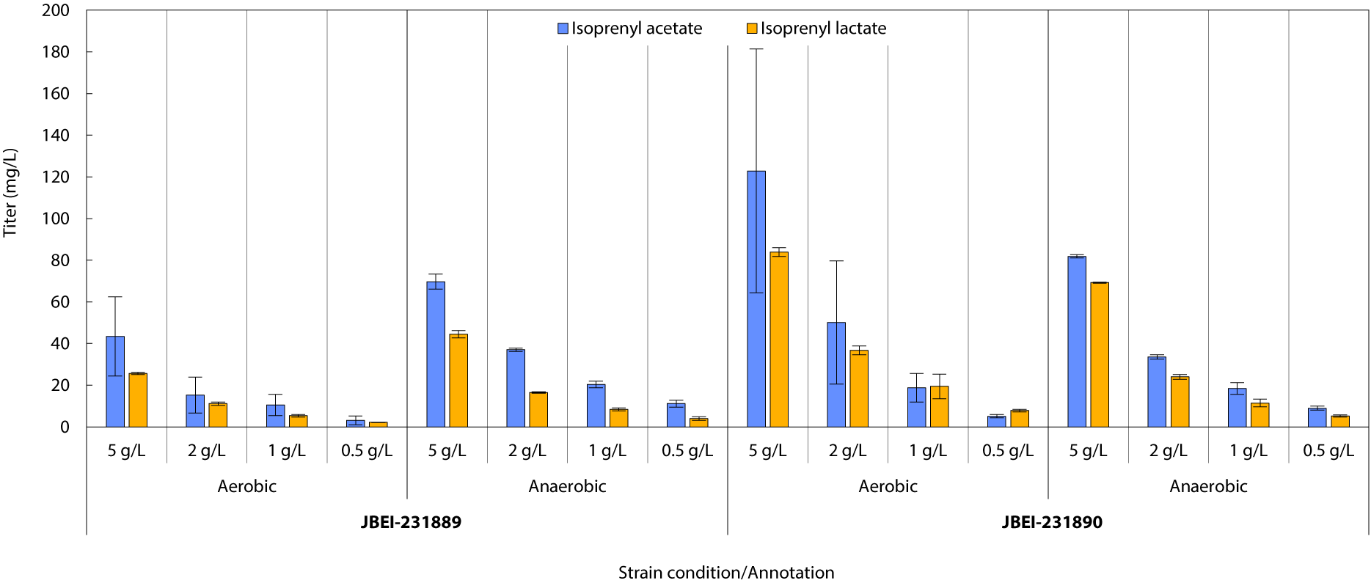
**

**Figure S7.** Ester production titrating isoprenol into high density cultures of *E. coli* DH1 harboring SAAT and either ldhA_Ec_ or ldh_Lm_ in 5 mL M9-MOPS media with 5 g/L yeast extract under aerobic and anaerobic conditions. No overlay was added, lending to variability of isoprenyl acetate titer between replicates.

**
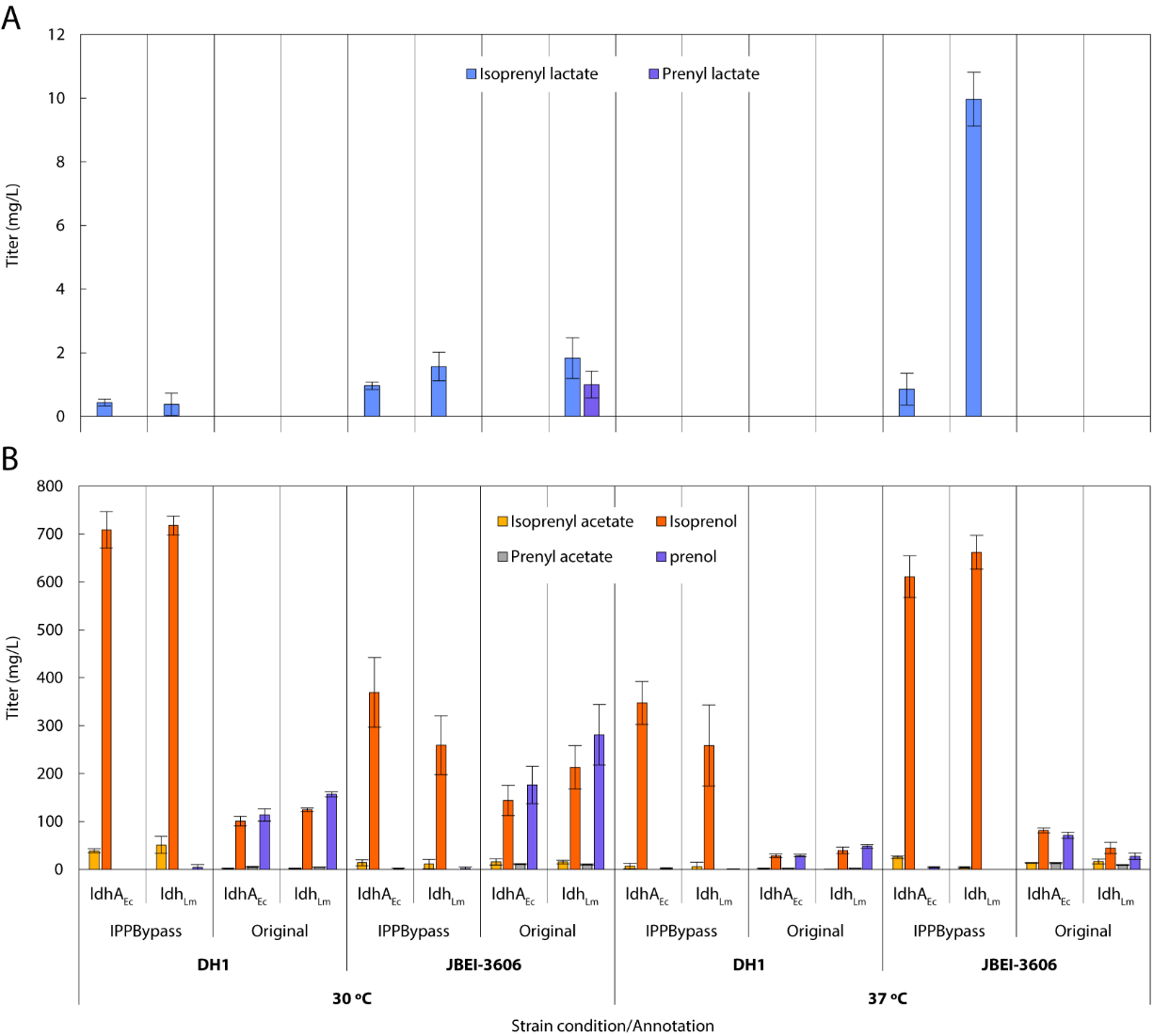
**

**Figure S8.** Small-scale (5 mL M9-MOPS media with 5 g/L yeast extract) production of lactate esters in DH1 and acetate pathway knockout strain JBEI-3606 at either (A) 30 °C or (B) 37 °C.

**
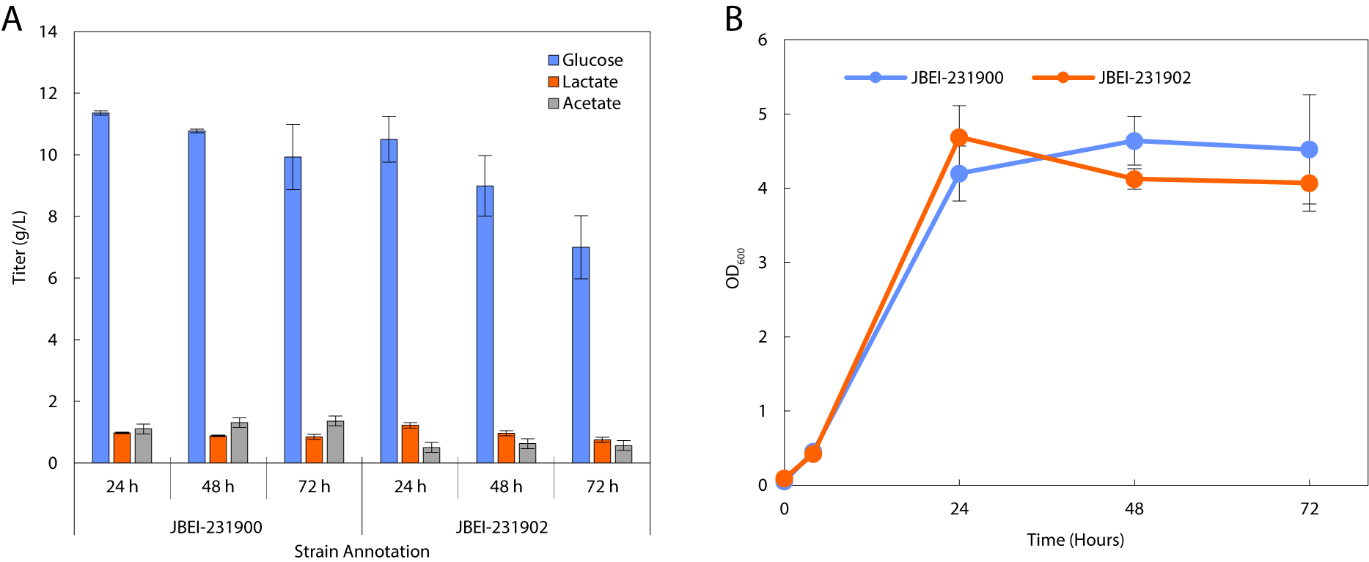
**

**Figure S9.** (A) Acetate, lactate, and glucose production by lactate ester strains as well as (B) OD_600_ growth data over 72 hours culturing.

**
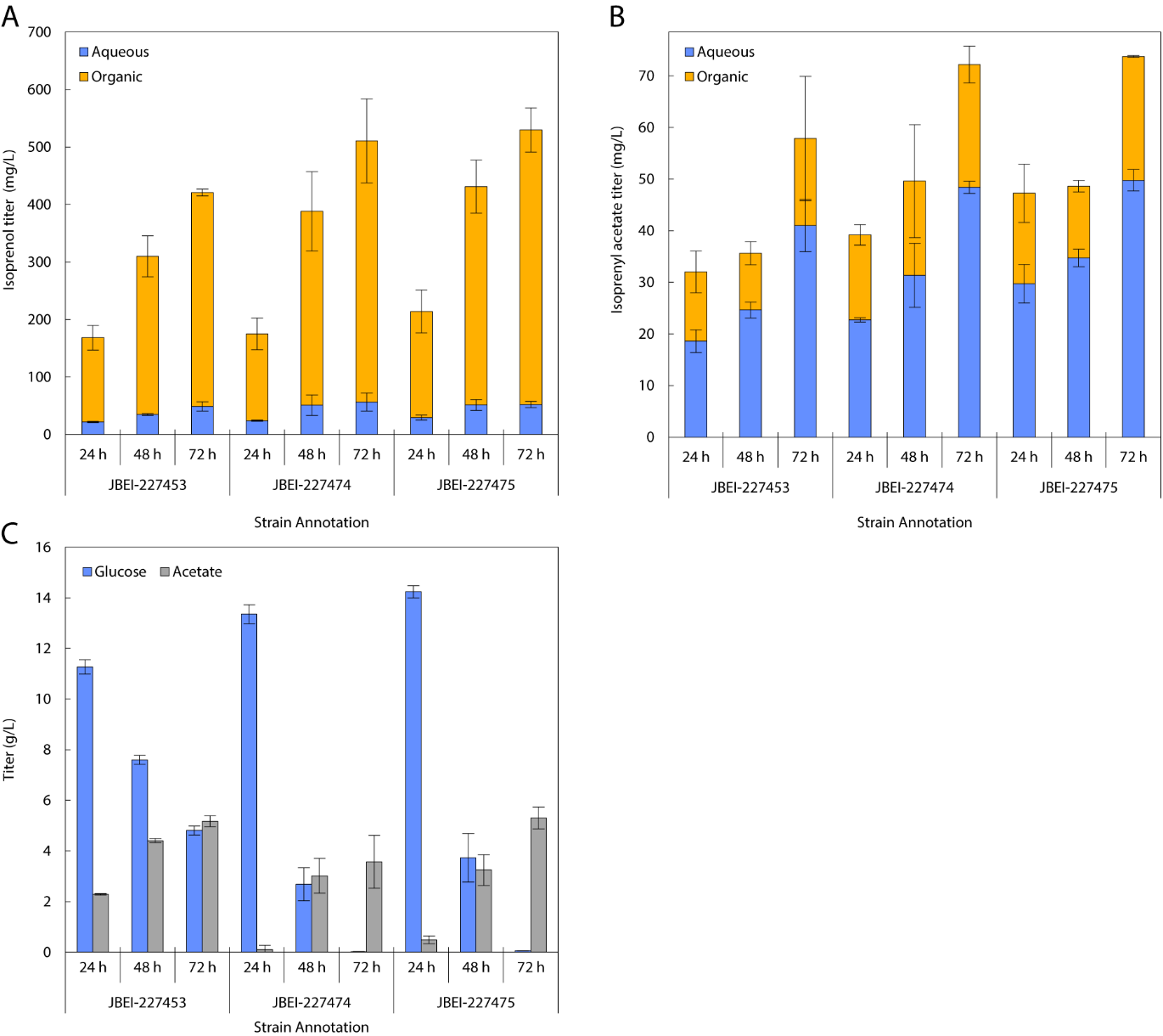
**

**Figure S10.** Production of isoprenol (A) and isoprenyl acetate (B) in strains harboring acetate pathway knockouts with *acs_Ec_* or *acs_Se_* in flask experiments. (C) *Acs* expression contributed to marginal changes in acetate titer over 72 hours.

**Theoretical yield calculations for isoprenyl acetate on glucose** [[2]](https://sciwheel.com/work/citation?ids=1310843&pre=&suf=&sa=0&dbf=0)**:**

*Overall stoichiometry:*

2C_6_H_12_O_6_ → C_7_H_12_O_2_ + 5CO_2_ + 12H

*Redox Balanced:*

2C_6_H_12_O_6_ + 2NADPH +8NAD^+^ → C_7_H_12_O_2_ + 5CO_2_ + 8NADH +2NADP^+^ + 6H^+^

Y_P_ = 1/2 = 0.5

Y_E_ = 24/36 = 0.6667

0.6667 *(128.17/180.156) = 0.4743 g/g

MVA pathway efficiency: 0.78

Maximum theoretical isoprenyl acetate yield via MVA on glucose: **0.3700 g/g**

**References:**

[1. Salis HM, Mirsky EA, Voigt CA. Automated design of synthetic ribosome binding sites to control protein expression. Nat Biotechnol. 2009;27:946–50.](https://sciwheel.com/work/bibliography/348364)

[2. Dugar D, Stephanopoulos G. Relative potential of biosynthetic pathways for biofuels and bio-based products. Nat Biotechnol. 2011;29:1074–8.](https://sciwheel.com/work/bibliography/1310843)
